# 26th Annual Computational Neuroscience Meeting (CNS*2017): Part 1

**DOI:** 10.1186/s12868-017-0370-3

**Published:** 2017-08-18

**Authors:** Sue Denham, Panayiota Poirazi, Erik De Schutter, Karl Friston, Ho Ka Chan, Thomas Nowotny, Dongqi Han, Sungho Hong, Sophie Rosay, Tanja Wernle, Alessandro Treves, Sarah Goethals, Romain Brette, Tomas Van Pottelbergh, Rodolphe Sepulchre, Alex D. Bird, Hermann Cuntz, Pedro J. Gonçalves, Jan-Matthis Lueckmann, Giacomo Bassetto, Marcel Nonnenmacher, Jakob H. Macke, Audrey J. Sederberg, Jason N. MacLean, Stephanie E. Palmer, Ulisse Ferrari, Christophe Gardella, Olivier Marre, Thierry Mora, Emina Ibrahimovic, Martin Müller, Jean-Pascal Pfister, Tushar Chauhan, Timothée Masquelier, Alexandre Montlibert, Benoit R. Cottereau, Moritz Helias, Jannis Schuecker, David Dahmen, Sven Goedeke, Alexandre Hyafil, Ainhoa Hermoso-Mendizabal, Pavel E. Rueda-Orozco, Santiago Jaramillo, David Robbe, Jaime de la Rocha, Marie Rooy, Fani Koukouli, David DiGregorio, Uwe Maskos, Boris Gutkin, Andrey Yu Verisokin, Darya V. Verveyko, Dmitry E. Postnov, Willy Wong, Omid Talakoub, Robert Chen, Milos Popovic, Dmitriy Lisitsyn, Eric Drebitz, Iris Grothe, Sunita Mandon, Andreas Kreiter, Udo Ernst, Peter A. Robinson, Xuelong Zhao, Kevin M. Aquino, John D. Griffiths, Grishma Mehta-Pandejee, Natasha Gabay, James MacLaurin, Somwrita Sarkar, Tim Kunze, Jens Haueisen, Thomas R. Knösche, Subutai Ahmad, Yuwei Cui, Marcus Lewis, Jeff Hawkins, Simona Olmi, Spase Petkoski, Fabrice Bartolomei, Maxime Guye, Viktor Jirsa, Hazem Toutounji, Daniel Durstewitz, Matteo Cantarelli, Adrian Quintana, Boris Marin, Matt Earnshaw, Padraig Gleeson, Robert Court, Robert McDougal, R. Angus Silver, Salvador Dura-Bernal, Stephen Larson, William W. Lytton, Giovanni Idili, Lorenzo Posani, Simona Cocco, Karel Ježek, Rémi Monasson

**Affiliations:** 1 School of Psychology, Faculty of Health & Human Sciences, Plymouth University, Plymouth, Devon, PL4 8AA UK; 2 Institute of Molecular Biology and Biotechnology (IMBB), Foundation for Research and Technology-Hellas (FORTH), Heraklion, Crete, Greece; 3 Okinawa Institute of Science and Technology Graduate University, Okinawa, Japan; 4 Wellcome Trust Centre for Neuroimaging, Institute of Neurology, UCL, London, WC1N 3BG UK; 5 School of Engineering and Informatics, University of Sussex, Brighton, BN1 9QJ UK; 6 Graduate School, Okinawa Institute of Science and Technology, Okinawa, 904-0495 Japan; 7 Computational Neuroscience Unit, Okinawa Institute of Science and Technology, Okinawa, 904-0495 Japan; 8 Cognitive Neuroscience Area, Scuola Internazionale Superiore di Studi Avanzati, Trieste, 34136 Italy; 9 Kavli Institute for Systems Neuroscience, NTNU, Trondheim, 7030 Norway; 10 Institut de la Vision, Sorbonne Universités, UPMC Univ Paris 06, INSERM, CNRS, Paris, 75012 France; 11 Department of Engineering, University of Cambridge, Cambridge, Cambridgeshire CB2 1PZ UK; 12 Ernst Strüngmann Institute for Neuroscience, Max Planck Society, Frankfurt, Germany; 13 Frankfurt Institute for Advanced Studies, Frankfurt, Germany; 14 Neural Systems Analysis, Research Center Caesar, An Associate of the Max Planck Society, Bonn, Germany; 15 Max Planck Institute for Biological Cybernetics, Tübingen, Germany; 16 Bernstein Center for Computational Neuroscience, Tübingen, Germany; 17 Present Address: Biomedical Engineering Department, Georgia Institute of Technology, Atlanta, GA 30306 USA; 18 Department of Neurobiology, University of Chicago, Chicago, IL 60637 USA; 19 Department of Organismal Biology and Anatomy, University of Chicago, Chicago, IL 60637 USA; 20 Committee on Computational Neuroscience, University of Chicago, Chicago, IL 60637 USA; 21 Institut de la Vision, Sorbonne Universités, INSERM, Paris, France; 22 Laboratoire de Physique Statistique, Ecole Normale Supérieure, Paris, France; 23 Institute of Molecular Life Sciences, University of Zürich, 8057, Zürich Switzerland; 24 Institute of Neuroinformatics, University of Zürich/ETH Zürich, 8057 Zürich, Switzerland; 25 Université de Toulouse, Centre de Recherche Cerveau et Cognition, Toulouse, France; 26 Centre National de la Recherche Scientifique, Toulouse Cedex, France; 27 Institute of Neuroscience and Medicine (INM-6) & Institute for Advanced Simulation (IAS-6), Jülich Research Centre, Jülich, Germany; 28 Department of Physics, Faculty 1, RWTH Aachen University, Aachen, Germany; 29 Idibaps, Barcelona, 08036 Spain; 30 Center for Brain and Cognition, Barcelona, 08001 Spain; 31 Universidad Nacional Autónoma de Mexico, Mexico City, Mexico; 32 Institute of Neuroscience, University of Oregon, Eugene, Oregon USA; 33 Inmed, Marseille, 13000 France; 34 Group for Neural Theory, Laboratoire de Neurosciences Cognitives, INSERM Unité 969, Département d’Études Cognitive, École Normale Supérieure, Paris, France; 35 Institut Pasteur, Neurobiologie intégrative des systèmes cholinergiques, Paris, France; 36 CNRS, UMR 3571, Paris, France; 37 Institut Pasteur, Dynamic Neuronal Imaging, Paris, France; 38 Centre for Cognition and Decision Making, National Research University Higher School of Economics, Moscow, Russia; 39 Department of Theoretical Physics, Kursk State University, Kursk, 305000 Russian Federation; 40 Department of Physics, Saratov State National Research University, Saratov, 410012 Russian Federation; 41 Department of Electrical and Computer Engineering, University of Toronto, Toronto, ON M5S3G4 Canada; 42 Institute of Biomaterials and Biomedical Engineering, University of Toronto, Toronto, ON M5S3G9 Canada; 43 Department of Biology, York University, Toronto, ON M3J1P3 Canada; 44 Krembil Research Institute - University Health Network, Toronto, ON M5T2S8 Canada; 45 Division of Neurology, Faculty of Medicine, University of Toronto, Toronto, ON M5S1A1 Canada; 46 Toronto Rehabilitation Institute - University Health Network, Toronto, ON M4G3V9 Canada; 47 Computational Neuroscience Lab, Institute for Theoretical Physics, Bremen University, Bremen, Germany; 48 Institute for Theoretical Neurobiology, Brain Research Institute, Bremen University, Bremen, Germany; 49 Ernst Strüngmann Institute (ESI) for Neuroscience, Frankfurt, Germany; 50 School of Physics, University of Sydney, Sydney, NSW 2006 Australia; 51 Center for Integrative Brain Function, University of Sydney, Sydney, NSW 2006 Australia; 52 Sir Peter Mansfield Imaging Center, University of Nottingham, Nottingham, UK; 53 Rotman Research Institute at Baycrest, Toronto, Ontario Canada; 54 Design Lab, School of Architecture, University of Sydney, Sydney, NSW 2006 Australia; 55 Max Planck Institute for Human Cognitive and Brain Sciences, Leipzig, Germany; 56 Institute of Biomedical Engineering and Informatics, Ilmenau University of Technology, Ilmenau, Germany; 57 Numenta, Redwood City, CA 94063 USA; 58 Weierstrass Institute, Mohrenstr. 39, 10117 Berlin, Germany; 59 Aix Marseille Université, Inserm, Institut de Neurosciences des Systèmes, UMR S 1106, 13005 Marseille, France; 60 Assistance Publique Hôpitaux de Marseille, Hôpital de la Timone, Service de Neurophysiologie Clinique, CHU, 13005 Marseille, France; 61 Faculté de Médecine de la Timone, centre de Résonance Magnétique et Biologique et Médicale (CRMBM,UMR CNRS-AMU 7339), Medical School of Marseille, Aix-Marseille Université, 13005 Marseille, France; 62 Department of Theoretical Neuroscience, Bernstein Center for Computational Neuroscience, Central Institute of Mental Health, Medical Faculty Mannheim, Heidelberg University, Heidelberg, Germany; 63 OpenWorm Foundation, Delaware, USA; 64 EyeSeeTea Ltd., London, UK; 65 Edinburgh University, Edinburgh, UK; 66 Department of Neuroscience, Physiology and Pharmacology, University College London, London, UK; 67 State University of New York Downstate Medical Center, Brooklyn, NY USA; 68 Yale University, New Haven, CT USA; 69 Laboratories of Statistical & Theoretical Physics, Ecole Normale Supérieure, Paris, France; 70 Laboratory of Experimental Neurophysiology, Biomedical Center, Charles University, Prague, Czech Republic

## K1 Auditory scene analysis: support and challenges for predictive coding

### Sue Denham

#### School of Psychology, Faculty of Health & Human Sciences, Plymouth University, Plymouth, Devon, PL4 8AA, UK

##### **Correspondence:** Sue Denham (S.Denham@plymouth.ac.uk)

*BMC Neuroscience* 2017, **18(Suppl 1)**:K1

Perception seems so simple. I look out of the window to see houses, trees, people walking past, the sky above, the grass below. I hear birds in the trees, cars going past, the distant sound of an alarm. The world is full of objects that make their presence known to me through my senses – what could be more simple? Yet the efficacy of perceptual experience hides a host of questions for which we do not yet have the answers. Information reaching our senses is generally incomplete, ambiguous, distributed in space and time and not neatly sorted according to its source, so a key function of our perceptual systems is to discover the likely causes of our sensations. Perception as inference or hypothesis testing, formalised in the predictive coding theory, offers an attractive framework for exploring these issues. From this perspective, regularities or patterns provide perceptual systems with some traction, allowing the formation of expectations and a basis for decomposing the world into discrete objects. But in the dynamic world which we inhabit, object representations must be similarly dynamic, and need to form and dissolve, dominate and yield, in a way that facilitates veridical perception. In this talk I will discuss auditory scene analysis in the context of predictive coding using experimental data, exemplar models, and the phenomenon of perceptual multistability.

## K2 Information coding with dendrites: Lessons from computational models

### Panayiota Poirazi

#### Institute of Molecular Biology and Biotechnology (IMBB), Foundation for Research and Technology-Hellas (FORTH), Heraklion, Crete, Greece

##### **Correspondence:** Panayiota Poirazi (poirazi@imbb.forth.gr)

*BMC Neuroscience* 2017, **18(Suppl 1)**:K2

The goal of this presentation is to provide a set of predictions generated by biophysical and/or abstract mathematical models regarding the role of dendrites in information processing, learning and memory across different brain regions. Towards this goal, I will present modelling studies from our lab –along with supporting experimental evidence- that investigate how dendrites may be used to facilitate the learning and coding of both spatial and temporal information at the single cell, the microcircuit and the neuronal network level. I will briefly present early work on how the dendrites of individual CA1 pyramidal neurons may allow a single cell to act as a 2-stage neural network classifier [1], thus massively increasing the storage capacity of the neural tissue [2]. I will then discuss how such dendritic nonlinearities may enable stimulus specificity in individual PFC pyramidal neurons during working memory [3] and underlie the emergence of sustained activity at the single cell and the microcircuit level [3, 4]. The role of dendrites in memory phenomena will be assessed using circuit models of the Dentate Gyrus implementing pattern separation [5, 6] as well as hippocampal models capable of learning associative memories and linking them across time [7]. This presentation aims to highlight how dendrites are likely to serve as key players in different memory functions.

**References**

1. Poirazi P, Brannon T, Mel BW: Pyramidal Neuron as 2-Layer Neural Network. *Neuron* 2003, 37: 989–999.

2. Poirazi P, Mel BW: Impact of Active Dendritic Processing and Structural Plasticity on Learning and Memory. *Neuron* 2001, 29: 779–796.

3. Sidiropoulou K, Poirazi P: Predictive features of persistent activity emergence in regular spiking and intrinsic bursting model neurons. *PLoS Comp. Biol.* 2012, 8(4): e1002489.

4. Papoutsi A, Sidiropoulou K, Poirazi P: Dendritic Nonlinearities Reduce Network Size Requirements and Mediate ON and OFF States of Persistent Activity in a PFC Microcircuit Model. *PLoS Comput. Biol.* 2014, 31: 10(7): e1003764.

5. Chavlis S, Petrantonakis P, Poirazi P: Dendrites of dentate gyrus granule cells contribute to pattern separation by controlling sparsity. *Hippocampus* 2017, 27(1): 89–110.

6. Danielson NB, Turi GF, Ladow M, Chavlis S, Petrantonakis PC, Poirazi P, Losonczy A: In Vivo Imaging of Dentate Gyrus Mossy Cells in Behaving Mice. Neuron 2017, 93(3):552–559.

7. Kastellakis G, Silva AJ, Poirazi P: Linking memories across time via neuronal and dendritic overlaps in model neurons with active dendrites. *Cell Reports* 2016, 17 (6): 1491–1504.

## K3 Molecular models of the early and late phases of bidirectional plasticity at cerebellar synapses

### Erik De Schutter

#### Okinawa Institute of Science and Technology Graduate University, Okinawa, Japan

##### **Correspondence:** Erik De Schutter (erik@oist.jp)

*BMC Neuroscience* 2017, **18(Suppl 1)**:K3

Synaptic plasticity at the parallel fiber to Purkinje cell synapse has been studied extensively, both experimentally and computationally. The initial focus was on long-term depression (LTD) evoked by concurrent parallel fiber and climbing fiber activation, but more recently experimental studies have emphasized the behavioral importance of long-term potentiation (LTP) triggered by exclusive parallel fiber activation. Expression of these forms of plasticity is based on changes in the number of AMPA receptors in the postsynaptic density (PSD), LTD leading to a decrease and LTP to an increase. As such, this plasticity is bidirectional and can be described as the outcome of a competition by opposing processes. Through studies of hippocampal plasticity, we have come to understand the importance of all aspects of the AMPA receptor cycle in bidirectional synaptic plasticity, with LTD increasing both diffusion out of the PSD and endocytosis of receptors and LTP favoring insertion of receptors that diffuse to the PSD. Moreover, the endosomal cycle is quite important because most endocytosed AMPA receptors are rapidly recycled to the postsynaptic membrane.

Calcium influx is always the first step in synaptic plasticity, but this influx is brief compared to the tens of minutes required to reach the maximum change in synaptic strength. For cerebellar LTD it is well established that the calcium signal activates a MAP-kinase based positive feedback loop that is essential for the early phase of LTD. We have built a completely new molecular model of bidirectional cerebellar plasticity that replicates experimental findings, including the dual role of nitric oxide in LTP and LTD. LTD requires activation of the MAP-kinase based positive feedback loop and this activation is controlled by CaM kinase. An emergent property of the model is an automatic shutdown of the positive feedback loop, corresponding to the end of the early phase.

In a second, simpler model, we have explored how the early phase can transition into a stable late phase by simple manipulations of the endosomal cycle. Unfortunately, experimental data on these processes is less complete, particularly about possible spatial restriction to single spines.

## K4 I am therefore I think

### Karl Friston

#### Wellcome Trust Centre for Neuroimaging, Institute of Neurology, UCL, London WC1N 3BG, UK

##### **Correspondence:** Karl Friston (k.friston@ucl.ac.uk)

*BMC Neuroscience* 2017, **18(Suppl 1)**:K4

This overview of the free energy principle offers an account of embodied exchange with the world that associates neuronal operations with actively inferring the causes of our sensations. Its agenda is to link formal (mathematical) descriptions of dynamical systems to a description of perception in terms of beliefs and goals. The argument has two parts: the first calls on the lawful dynamics of any (weakly mixing) ergodic system – from a single cell organism to a human brain. These lawful dynamics suggest that (internal) states can be interpreted as modelling or predicting the (external) causes of sensory fluctuations. In other words, if a system exists, its internal states must encode probabilistic beliefs about external states. Heuristically, this means that if I exist (am) then I must have beliefs (think). The second part of the argument is that the only tenable beliefs I can entertain about myself are that I exist. This may seem rather obvious; however, it transpires that this is equivalent to believing that the world – and the way it is sampled – will resolve uncertainty about the causes of sensations. We will consider the implications for functional anatomy, in terms of predictive coding and hierarchical architectures, and conclude by looking at the epistemic behaviour that emerges – using simulations of active inference.

## F1 Mixture processing in a biophysical model of the early olfactory system of honeybees

### Ho Ka Chan, Thomas Nowotny

#### School of Engineering and Informatics, University of Sussex, Brighton, BN1 9QJ, UK

##### **Correspondence:** Ho Ka Chan (hc338@sussex.ac.uk)

*BMC Neuroscience* 2017, **18(Suppl 1)**:F1

In their natural environment, Insects often encounter complex mixtures of odors in their natural environment. It is an important open question whether and how the processing of complex mixtures of multi-component odors differs from that of simpler mixtures or single components. To approach this question, we built a full-size model of the early olfactory system of honeybees, which predicts responses to both single odorants and mixtures. The model is designed so that olfactory response patterns conform to the statistics derived from experimental data [1] for a variety of its properties. It also takes into account several biophysical processes at a minimal level, including processes of chemical binding and activation in receptors, and spike generation and transmission in the antennal lobe network. We verify that key findings from other experimental data not used in building the model [2–4] are reproduced in it. In particular, we replicate the strong correlation among receptor neurons and the weaker correlation among projection neurons observed in experimental data [1, 2] and show that this decorrelation is predominantly due to inhibition by interneurons. By simulation and mathematical analysis of our model, we demonstrate that the chemical processes of receptor binding and activation already lead to significant differences between the responses to mixtures and those to single component stimuli. On average, the response latency of olfactory receptor neurons at low stimulus concentrations is reduced (see Fig. 1a) and the response patterns become less variable across concentrations (see Fig. 1b) as the number of odor components in the stimulus increases. These effects are preserved in the projection neurons. Our results provide hints that the early olfactory system in insects may be particularly efficient in processing mixtures, which corresponds well to the observation that chemical signaling in nature predominantly utilizes mixtures.
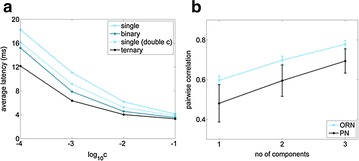


**Fig. 1 a** The average response latency decreases with the number of components in the odor stimulus. The effect is most significant when the stimulus concentration is low. **b** The pairwise correlation, averaged over all ORNs, between the response patterns at low and high concentration increases with the number of components in the odor stimulus

**Acknowledgements**

This work was supported by HFSP, RGP0053/2015 and EPSRC, EP/J019690/1.

**References**

1. Galizia CG, Sachse S, Rappert A, Menzel R. The glomerular code for odor representation is species specific in the honeybee Apis mellifera. *Nat Neurosci.* 1999 May, 2(5):473–478.

2. Ditzen M. Odor concentration and identity coding in the antennal lobe of the honeybee Apis mellifera [PhD thesis]. Berlin: Freie Universität Berlin; 2005.

3. Szyszka P, Gerkin RC, Galizia CG, Smith BH. High-speed odor transduction and pulse tracking by insect olfactory receptor neurons. *Proc Natl Acad Sci U S A*. 2014, 111(47):16925–16930.

4. Deisig N, Giurfa C, Sandoz JC. Antennal lobe processing increases separability of odor mixture representations in the honeybee. *J. Neruophysiol* 2010, 103:2185–21

## F2 Heterogeneous layers stabilize propagation of a multiplexed spike signal in a feedforward network

### Dongqi Han^1^, Sungho Hong^2^

#### ^1^Graduate School, Okinawa Institute of Science and Technology, Okinawa, 904–0495, Japan; ^2^Computational Neuroscience Unit, Okinawa Institute of Science and Technology, Okinawa, 904–0495, Japan

##### **Correspondence:** Dongqi Han (dongqi.han@oist.jp)

*BMC Neuroscience* 2017, **18(Suppl 1)**:F2

Feedforward networks are ubiquitous structures in neural systems and have been studied in many contexts such as models for signal transmission [1, 2], architectures for rich information processing [3], etc. However, most studies have ignored an important property commonly observed in real feedforward networks: neurons in one layer have contrasting characteristics from those in other layers. For example, the cerebellar granule cells are tiny and relatively simplistic neurons while their postsynaptic targets, the Purkinje cells, are much bigger, complex, and therefore have very different intrinsic properties. What would be the role of such layer-to-layer differences in neural circuits?

Here we address this question by simulation of a model feedforward network, inspired by a recent experimental study on the *Drosophila* olfactory system [4]. In this model, all the adjacent layers have Morris-Lecar neurons with different excitability types from each other and therefore different computational functions. If one layer has cells with class I excitability, which behave like integrators of inputs, neurons in the adjacent layer are of class III, which act as coincidence detectors [5], and vice versa.

We found that spikes from one layer evoked a response in next layer neurons better when they had the same excitability type. However, in a deep feedforward network, this caused gradual accumulation of signal distortion, leading to the undesirable responses in deep layers that all the neurons either fired synchronously or became silent, as seen in classical studies (e.g., [1]). On the other hand, the network with heterogeneous layers demonstrated a novel signal transformation property as observed in [4] (Fig. 1a), and showed stable propagation of a signal into deep layers with a preserved temporal fidelity and spike count (Fig. 1b, c). We analyzed the result by using a phase space method in [1] and showed how mixing different coding schemes enables this feature (Fig. 1c). We conclude that heterogeneous layers in feedforward neural networks can be a mechanism for optimal information transfer.
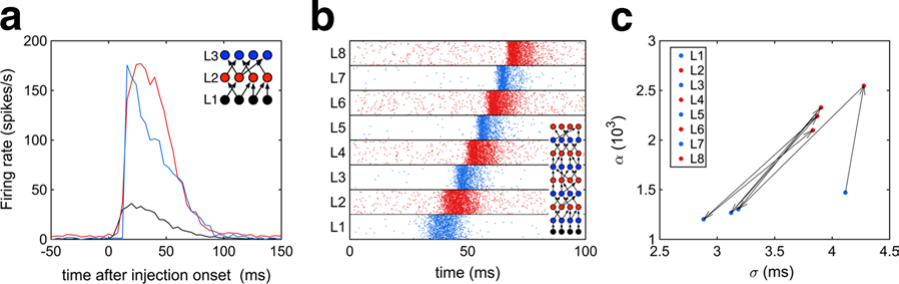


**Fig. 1 a** Firing rate of input (black, L1), integrator (red, L2), and coincidence detector (blue, L3) neurons. Note that a peak of L3 firing precedes that of L2, as observed in [4]. **b** Stable spike propagation in a network with many heterogeneous layers. **c** Layer-to-layer change in the SD of spike times (σ) and spike count (α) of **b**

**References**

1. Diesmann M, Gewaltig M-O, Aertsen A. Stable propagation of synchronous spiking in cortical neural networks. *Nature* 1999, 402:529–33.

2. Kumar A, Rotter S, Aertsen A. Spiking activity propagation in neuronal networks: reconciling different perspectives on neural coding. *Nat Rev Neurosci.* 2010, 11:615–27.

3. Serre T, Oliva A, Poggio T. A feedforward architecture accounts for rapid categorization. *PNAS* 2007, 104:6424–9.

4. Jeanne JM, Wilson RI. Convergence, Divergence, and Reconvergence in a Feedforward Network Improves Neural Speed and Accuracy. *Neuron* 2015, 88:1014–26.

5. Ratté S, Hong S, De Schutter E, Prescott SA. Impact of neuronal properties on network coding: roles of spike initiation dynamics and robust synchrony transfer. *Neuron* 2013, 78:758–72.

## F3 Modeling grid fields instead of modeling grid cells

### Sophie Rosay^1^, Tanja Wernle^2^, Alessandro Treves^1^

#### ^1^Cognitive Neuroscience Area, Scuola Internazionale Superiore di Studi Avanzati, Trieste, 34136, Italy; ^2^Kavli Institute for Systems Neuroscience, NTNU, Trondheim, 7030, Norway

##### **Correspondence:** Sophie Rosay (srosay@sissa.it)

*BMC Neuroscience* 2017, **18(Suppl 1)**:F3

Grid cells are neurons found in the rodent medial entorhinal cortex. They take their name from their astonishing firing patterns: grid cells are active specifically in certain regions of physical space, called grid fields, that form a triangular grid tesselating the space explored by the animal. While experiments have been investigating the geometrical properties of grid fields [1], computationalists have tried to explain them by neural network models. However, these models still fail to account for some of the experimental results, in particular how two distinct grid patterns are integrated when two compartments are merged into one (Wernle et al, in preparation).

We take a different approach: instead of modelling grid cells, we directly model single grid fields as point objects interacting with each other and with the environment’s borders (see Fig. 1a). This description is motivated by the way grid patterns are naturally considered as geometrical objects. We thus consider a system of interacting objects evolving as colloidal particles on a substrate [2]. First, we consider only grid fields from one grid cell, then we add coupling between several cells. We simulate the system with varying forms and intensity of the interactions. The simplicity of the model allows us to test many such possibilities and their outcome in several ‘experimental’ setups.

We show that under certain conditions the model does reproduce the behavior of experimental grid fields (see Fig. 1b). These conditions imply repulsion between fields, the involvement of a large number of fields, interaction between grid cells and with the walls. We are able to reproduce observed data from experiments in merged environments. We can also make predictions for setups not tested experimentally yet.

The question that then naturally arises is how to connect our description at the level of grid fields to models at the level of grid cells. We show how our grid-field model puts constraints on models of the underlying grid cells. Conversely, we discuss how existing grid-cell models can be described at the level of grid fields.
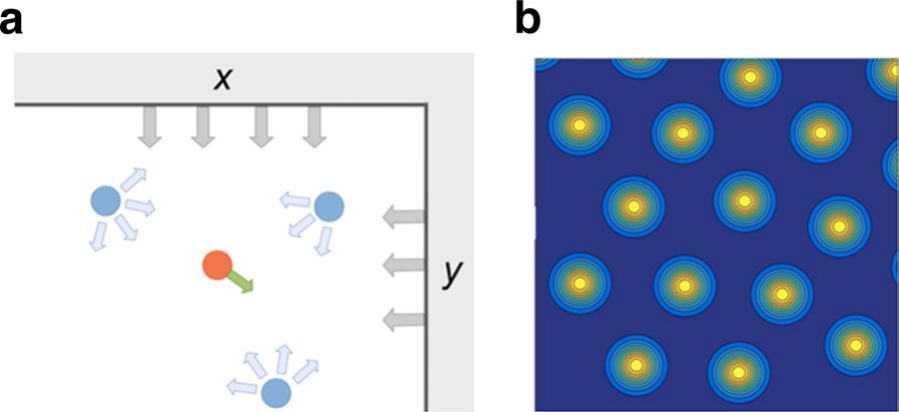


**Fig. 1** General idea of grid field modeling. **a** Schematic representation of the model. A given grid field (*red*) feels the influence of other grid fields (*blue*) as well as the borders (*grey*) plus a viscosity force (*green*). **b** Example of a resulting pattern in a square box, converted into a firing rate map

In conclusion, tackling the issue of grid patterns from a grid-field perspective provides new insights on their formation. Beyond grid cells, our work raises the question of the ultimate purpose of a model and the subtle interplay between description and explanation.

**References**

1. Stensola T, Stensola H, Moser MB, Moser EI: Shearing-induced asymmetry in entorhinal grid cells. Nature 2015, 518(7538): 207–212.

2. Manoharan VN: Colloidal matter: Packing, geometry, and entropy. *Science* 2015, 349(625): 1253751.

## O1 Impact of axon initial segment geometry on excitability

### Sarah Goethals, Romain Brette

#### Institut de la Vision, Sorbonne Universités, UPMC Univ Paris 06, INSERM, CNRS, Paris, 75012, France

##### **Correspondence:** Romain Brette (romain.brette@inserm.fr)

*BMC Neuroscience* 2017, **18(Suppl 1)**:O1

In most vertebrate neurons, action potentials are triggered at the distal end of the axon initial segment (AIS). They are then transmitted to the soma where they are regenerated and further propagated in the dendritic tree. The AIS position and length can be altered by changes in electrical activity, suggesting a strong link between AIS geometry and excitability. We studied theoretically the influence of AIS geometry on the somatic threshold for AP initiation. For this purpose, we solved the cable equation with appropriate boundary conditions in a cylindrical axon model. Our theoretical analysis shows that the somatic threshold depends logarithmically on the surfacic sodium conductance density and that increasing either the AIS length or the AIS start position lowers the threshold. We confirmed our prediction with numerical simulations in a more detailed neuron model. Our analysis suggests that either a longer or a more distal AIS increases excitability, which supports a current hypothesis that the AIS is preferably isolated from the large capacitance of the soma. Secondly, we examined how the AIS geometry influences the peak axonal current that is transmitted to the soma at spike initiation. Again, we used cable analysis to study this current in a two-cylinder model that represents the main geometrical features of a thick-tufted layer 5 pyramidal neurons. Our analysis shows that in order to obtain somatic spikes with a given speed, the AIS position should be proportional to the diameter of the apical dendrite raised to the -3/2. We confirmed this theoretical result with numerical simulations of a more detailed model. In addition, correlation analysis of layer 5 pyramidal neurons morphology confirms this theoretical prediction [1]. Our previous analyses suggest that the AIS geometry is finely tuned for successful spike transmission to the soma. More generally, neural systems tend to be efficient in their use of resources [2], which suggests that AIS geometry might also be optimized for minimal energy consumption. As the energy consumption at subthreshold voltages is proportional to the number of channels, we asked whether there exists an AIS geometry that minimizes the total number of sodium channels. For this purpose, we used variational techniques to calculate the AIS geometry that minimizes total Na conductance, for a given spike threshold and axonal current.

**Conclusion**

Our theoretical analysis shows that AIS geometry has a strong impact on several aspects of excitability including energy consumption, which suggests that the AIS morphology is functionally tuned and possibly optimized.

**References**

1. Hamada MS, Goethals S, de Vries SI, Brette R, Kole MH: Covariation of axon initial segment location and dendritic tree normalizes the somatic action potential. *Proceedings of the National Academy of Sciences* 2016, 113(51):14841–14846.

2. Niven JE, Laughlin SB: Energy limitation as a selective pressure on the evolution of sensory systems. *Journal of Experimental Biology* 2008, 211(11):1792–1804.

## O2 Can integrate-and-fire models simulate robust neuromodulation?

### Tomas Van Pottelbergh, Rodolphe Sepulchre

#### Department of Engineering, University of Cambridge, Cambridge, Cambridgeshire, CB2 1PZ, UK

##### **Correspondence:** Tomas Van Pottelbergh (tmjv2@cam.ac.uk)

*BMC Neuroscience* 2017, **18(Suppl 1)**:O2

By controlling the state of neuronal populations, neuromodulators ultimately affect behaviour. A key neuromodulation mechanism is the alteration of neuronal excitability via the modulation of ion channel expression. This type of neuromodulation is normally studied via conductance-based models, but those models are computationally challenging for large-scale network simulations needed in population studies. Integrate-and-fire models provide a computationally advantageous alternative, but such models are only partially successful in robustly capturing modulation between firing patterns.

In this work, we propose a modelling framework that extracts the qualitative properties of neuromodulation to produce neuromodulable and computationally efficient neuron models. Our framework is based on dynamic I-V curves, i.e. instantaneous I-V curves in a certain timescale [1]. These dynamic I-V curves make the connection between qualitative conductance-based models and integrate-and-fire models: how a change in ion channel conductance can be related to a change of dynamic I-V curves and subsequently to a parameter change in the reduced integrate-and-fire model. We focus on the modulation between tonic firing and bursting as an example. We argue that this modulation crucially relies on the co-regulation of two points of high sensitivity (i.e. excitability) in two distinct timescales. The points of high sensitivity are local extrema in the I-V curves and correspond to an exact balance of positive and negative feedback. Those signatures have a direct correlate in the fast-slow phase portraits: a hysteretic V-nullcline in the presence of one (fast) balance, and a mirrored hysteresis in the presence of both a fast and a slow balance [2]. The classical quadratic integrate-and-fire model captures the fast balance, but does not account for the slow one.

The simple idea underlying the proposed multi-quadratic integrate-and-fire model (MQIF) is to allow for several distinct balance points in an integrate-and-fire model. An integrate-and-fire model with two balance points is shown to robustly capture the neuromodulation between spiking and bursting, opening novel computational avenues for large-scale simulation of neuromodulated populations. The robustness and modulation properties of this integrate-and-fire model are in sharp contrast to those of existing (generalised) integrate-and-fire models, which lack the slow excitability.

**References**

1. Drion G, Franci A, Dethier J, Sepulchre R: Dynamic Input Conductances Shape Neuronal Spiking. *eNeuro* 2015, 2(1):ENEURO.0031-14.2015.

2. Franci A, Drion G, Sepulchre R: An Organizing Center in a Planar Model of Neuronal Excitability. *SIAM J Appl Dyn Syst* 2012, 11(4):1698–1722.

## O3 Sholl analysis predicted by dendrite spanning fields

### Alex D Bird^1,2^, Hermann Cuntz^1,2^

#### ^1^Ernst Strüngmann Institute for Neuroscience, Max Planck Society, Frankfurt, Germany; ^2^ Frankfurt Institute for Advanced Studies, Frankfurt, Germany

##### **Correspondence:** Alex D Bird (alex.neurosci@gmail.com)

*BMC Neuroscience* 2017, **18(Suppl 1)**:O3

Sholl analysis has been an important technique in dendritic anatomy for over sixty years [1]. In counting the number of dendritic branches at a given distance from the soma, the Sholl intersection profile is often taken as a crucial measure of dendritic complexity; it has been used in a broad range of applications, from estimating the expected number of possible synapses [2], to evaluating the changes in structure induced by pathologies [3].

We have shown that Sholl intersection profiles can be predicted by two more basic measures: the domain spanned by the dendritic arbor and the angular distribution of how far dendritic segments deviate from a direct path to the soma (Fig. 1c). The first measure is principally determined by axon location and hence microcircuit structure [4]; the second arises from optimal wiring [5]. These two measures allow Sholl analysis to be given a more functional interpretation across all of its applications.
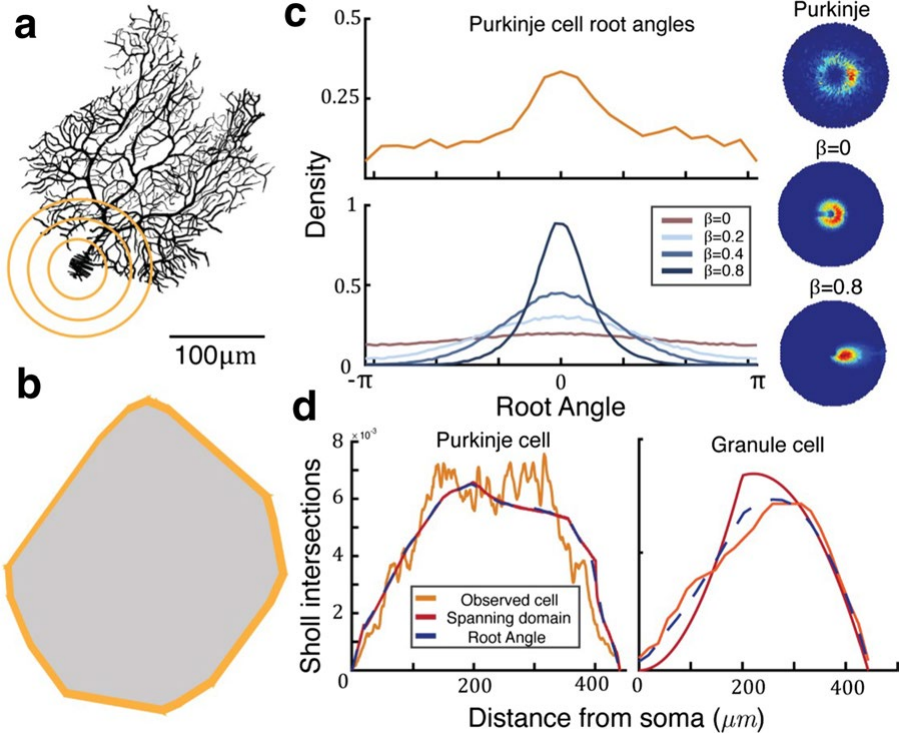


**Fig. 1** The dendrite spanning field predicts the Sholl intersection profile of a Purkinje cell. **a** Rat Purkinkje cell [6] and schematic of Sholl analysis: the number of times the dendrite intersects with a gold arc gives the value of the Sholl intersection profile at that radius. **b** The spanning field of the above cell. **c** Root angle distributions and (inset) joint angular and Euclidean connection probabilities for the Purkinje cell (*top*) and artificial dendrites with different balances **ß** between wiring and delay costs (*bottom*). **d** Sholl intersection profiles for rat Purkinje cell (*left*) and mouse dentate gyrus granule cell [7] (*right*): observed (*gold*), predicted by just the spanning field (*red*), and predicted by the spanning field and root angles (*blue dashed*)

**References**

1. Sholl D: Dendritic organisation in the neurons in the visual and motor cortices of the cat. *J Anat* 1953, 87(4):387–406.

2. Liley D & Wright J: Intracortical connectivity of pyramidal and stellate cells: estimates of synaptic densities and coupling symmetry. *Comp Neur Sys* 1994, 5(2):175–189.

3. Williams P, Thirgood R, Oliphant H *et al*: Retinal ganglion cell dendritic degeneration in a mouse model of Alzheimer’s disease. *Neurobiol Aging* 2013, 34(7):1799–1806.

4. Stepanyants A & Chklovskii D: Neurogeometry and potential synaptic connectivity. *Trends Neurosci* 2005, 28(7): 387–394.

5. Cuntz H, Forstner F, Borst A, & Häusser M: One rule to grow them all: A general theory of neuronal branching and its practical application. *PLoS Comp Bio* 2010, 6(8):e1000877.

6. Vetter P, Roth A, & Häusser M: Propagation of action potentials in dendrites depends on dendritic morphology. *J Neurophysiol* 2001, 85(2): 926–937.

7. Schmidt-Hieber C, Jonas P, & Bischofberger J. Dendritic signal processing and coincidence detection in dentate gyrus granule cells. *J Neurosci* 2007, 27(31):8430–8441.

## O4 Flexible Bayesian inference for complex models of single neurons

### Pedro J Gonçalves^1^, Jan-Matthis Lueckmann^1^, Giacomo Bassetto^1^, Marcel Nonnenmacher^1^, Jakob H Macke^1,2,3^

#### ^1^Neural Systems Analysis, research center Caesar, an associate of the Max Planck Society, Bonn, Germany; ^2^Max Planck Institute for Biological Cybernetics, Tübingen, Germany; ^3^Bernstein Center for Computational Neuroscience, Tübingen

##### **Correspondence:** Pedro J Gonçalves (pedro.goncalves@caesar.de)

*BMC Neuroscience* 2017, **18(Suppl 1)**:O4

Characterizing the input-output transformations of single neurons is critical for understanding neural computation. Single-neuron models have been extensively studied, ranging from simple phenomenological models to complex multi-compartment neurons. However, linking mechanistic models of single-neurons to empirical observations of neural activity has been challenging. Statistical inference is only possible for a few neuron models (e.g. GLMs), and no generally applicable, effective statistical inference algorithms are available: As a consequence, comparisons between models and data are either qualitative or rely on manual parameter tweaking, parameter-fitting using heuristics or brute-force search [1]. Furthermore, parameter-fitting approaches typically return a single best-fitting estimate, but do not characterize the entire space of models that would be consistent with data (the ‘posterior distribution’).

We overcome this limitation by presenting a general method to infer the posterior distribution over model parameters given observed data on complex single-neuron models. Our approach can be applied in a `black box’ manner to a wide range of single-neuron models without requiring model-specific modifications. In particular, it extends to models without explicit likelihoods (e.g. most single-neuron models). We achieve this goal by building on recent advances in likelihood-free Bayesian inference [2]: the key idea is to simulate multiple data-sets from different parameters, and then to train a probabilistic neural network which approximates the mapping from data to posterior distribution.

We illustrate this approach using single- and multi-compartment models of single neurons: On simulated data, estimated posterior distributions recover ground-truth parameters, and reveal the manifold of parameters for which the model exhibits the same behaviour. On in-vitro recordings of membrane voltages, we recover multivariate posteriors over biophysical parameters, and voltage traces accurately match empirical data. Our approach will enable neuroscientists to perform Bayesian inference on complex neuron models without having to design model-specific algorithms, closing the gap between biophysical and statistical approaches to single-neuron modelling.

**References**

1. Druckmann S, Banitt Y, Gidon A, Schurmann F, Markram H, Segev I: A novel multiple objective optimization framework for constraining conductance-based neuron models by experimental data. *Front Neurosci* 2007, 1(1):7–18.

2. Papamakarios G, Murray I: Fast ε-free inference of simulation models with Bayesian conditional density estimation. In *Advances in Neural Information Processing Systems* 2016, pages 1028–1036.

## O5 Learning to read out predictive information in early visual processing

### Audrey J Sederberg^1,2,3^, Jason N MacLean^2,4^, Stephanie E Palmer^3,4^

#### ^1^Biomedical Engineering Department, Georgia Institute of Technology, Atlanta, GA 30306, USA (current address); ^2^Department of Neurobiology, University of Chicago, Chicago, IL, 60637, USA; ^3^Department of Organismal Biology and Anatomy, University of Chicago, Chicago, IL, 60637, USA; ^4^Committee on Computational Neuroscience, University of Chicago, Chicago, IL 60637, USA

##### **Correspondence:** Audrey J Sederberg (audrey.sederberg@gatech.edu)

*BMC Neuroscience* 2017, **18(Suppl 1)**:O5

To generate appropriate behavior, the brain must predict the future state of the world from past sensory information. Taking the salamander visual system as an example, at the minimum such predictions need to compensate for the 50–80 ms processing time of the retina [1] as well as the time for a motor response to be generated. Making these predictions requires leveraging the spatiotemporal structure of the natural world, a computation that is performed efficiently at the first stage of visual processing, in populations of retinal ganglion cells [2]. Neurons downstream of the retina infer predictions about object motion from the firing of their inputs, but to do so, downstream cells must learn to read out predictive information from the retinal activity.

More concretely, stimulus predictive information in a sensory population is defined as the mutual information of particular patterns of spiking across retinal ganglion cells (RGCs) with the future stimulus [2]. We consider the output of a downstream model neuron that receives weighted inputs from several RGCs. By constructing moving-bar dynamics that contain both predictable and stochastic motion, we can visualize the predictive information in readout spiking as the difference between the prior stimulus distribution (Fig. 1a, gray) and the spike-triggered stimulus distribution: the larger the difference, the more informative the spike. In this example, the readout neuron was informative of the future stimulus (Fig. 1a), but most other readouts were not (not shown). Even for an experimenter with a well-controlled sensory input, finding this optimal readout in the space of all possible readouts is difficult. In more realistic circumstances, the organism must make predictions about the future state of complex natural stimuli from the retinal spiking activity alone (Fig. 1b).

Here we address whether biologically plausible learning rules can find readout weights that transform RGC input into predictive downstream output. Input activity from the RGCs was previously recorded in the salamander retina in response to a natural movie (Fig. 1b). We first show that internal predictive information, the information that the readout has about its own future input, is correlated with stimulus predictive information, so that becoming more predictive of its inputs drives the readout neuron to encode more information about the future stimulus. Starting from a set of random weights connecting the RGCs to the readout neuron, we allow the weights to evolve via spike timing-dependent plasticity. Across many groups of RGCs, we find that learned readouts convey 80% of the possible predictive information for groups of four cells, but only 30% for groups of ten cells. This decrease reflects a compressibility limit of predictive information and suggests an optimal pooling size for cells downstream from retina.
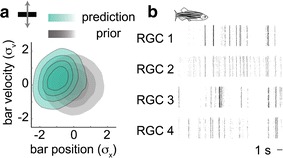


**Fig. 1 a** Spike-triggered average of future stimulus position and velocity for a particular readout of population spiking activity with high predictive information. **b** Raster plots for simultaneously recorded retinal ganglion cells (RGCs) in response to a naturalistic movie featuring swimming fish. These are used to drive learning simulations

**Acknowledgements**

AJS was supported by the Mary-Rita Angelo Fellowship. SEP was supported by the Alfred P. Sloan Foundation. JNM was supported by National Science Foundation CAREER Award No. 095286.

**References**

1. Segev R, Puchalla J, Berry MJ II: Functional organization of ganglion cells in the salamander retina. *J Neurophysiol* 2006, 95: 2277–2292.

2. Palmer SE, Marre O, Berry MJ II, Bialek WB: Predictive information in a sensory population. *PNAS* 2015, 112: 6908–6913.

## O6 Closed-loop estimation of retinal network sensitivity reveals signature of efficient coding

### Ulisse Ferrari^1^, Christophe Gardella^1,2^, Olivier Marre^1^, Thierry Mora^2^

#### ^1^Institut de la Vision, Sorbonne Universités, INSERM, Paris, France; ^2^Laboratoire de Physique Statistique, Ecole Normale Supérieure, Paris, France

##### **Correspondence:** Ulisse Ferrari (ulisse.ferrari@gmail.com)

*BMC Neuroscience* 2017, **18(Suppl 1)**:O6

According to the theory of efficient coding, sensory systems are adapted to represent natural scenes with high fidelity and at minimal metabolic cost. Testing this hypothesis for sensory structures performing non-linear computations on high dimensional stimuli is still an open challenge. Here we develop a method to characterize the sensitivity of the retinal network to perturbations of a stimulus. Using closed-loop experiments, we explore selectively the space of possible perturbations around a given stimulus. We then show that the response of the retinal population to these small perturbations can be described by a local linear model. Using this model, we computed

the sensitivity of the neural response to arbitrary temporal perturbations of the stimulus, and found a peak in the sensitivity as a function of the frequency of the perturbations. Based on a minimal theory of sensory processing, we argue that this peak is set to maximize information transmission. Our approach is relevant to testing the efficient coding hypothesis locally in any context where no reliable encoding model is known.

**Acknowledgements**

This work was supported by ANR TRAJECTORY, ANR OPTIMA, the French State program Investissements d’Avenir managed by the Agence Nationale de la Recherche [LIFESENSES: ANR-10-LABX-65], European Comission grant from the Human Brain Project n. FP7-604102, and National Institutes of Health grant n. U01NS090501.

## O7 Learning Quantal Parameters through Expectation-Maximization

### Emina Ibrahimovic^1,2^, Martin Müller^1^, Jean-Pascal Pfister^2^

#### ^1^Institute of Molecular Life Sciences, University of Zürich, 8057, Zürich, Switzerland; ^2^Institute of Neuroinformatics, University of Zürich/ETH Zürich, 8057, Zürich, Switzerland

##### **Correspondence:** Emina Ibrahimovic (emina.ibrahimovic@imls.uzh.ch)

*BMC Neuroscience* 2017, **18(Suppl 1)**:O7

Large synapses, i.e. the neuromuscular junction (NMJ) or the calyx of Held, have been invaluable model synapses that have significantly advanced the field of synaptic transmission. No generative model approach can faithfully retrieve quantal parameters from synapses with a large number of release sites (N). Here we propose an expectation maximization (EM) method that is based on particle smoothing (PS) to extract quantal parameters from large N synapses. In contrast to an existing EM-based approach [1], using Baum-Welch (BW) which scales with a complexity of N^4 and hence cannot retrace quantal parameters of synapses with hundreds of release sites, our method is independent of N and therefore suitable for large synapses. First, our model was validated on synthetic data. As shown in Fig. 1, all parameters θ = {N,p,q,σ,τ} were faithfully retrieved. Next, we applied the model to the *Drosophila* NMJ, which is predicted to harbor hundreds of release sites. The model predicted quantal parameters that are in line with parameters predicted by two empirical approaches (variance-mean analysis and cumulative amplitude analysis of postsynaptic currents). In contrast to these two techniques, which require data recorded under conditions of high release probability (p), our method is independent of p or stimulation protocol. Given the genetic tractability of this synapse, our theoretical approach is expected to help linking quantal parameters to molecular function.
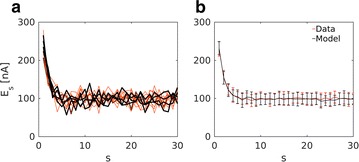


**Fig. 1 a** Postsynaptic currents at the *Drosophila* NMJ, 50 trains of 30 presynaptic action potentials at 60 Hz compared with model generated data from the fitted parameters at each stimulation step s; number of release sites N = 710, release probability p = 0.44, quantal content q = 0.74 nA, background noise σ = 13.22 nA and refilling time constant τ = 59 ms. **b** Comparison of the mean and standard deviation

**Reference**

1. Alessandro Barri, Yun Wang, David Hansel, Gianluigi Mongillo, Quantifying Repetitive Transmission at Chemical Synapses: A Generative-Model Approach, *eNeuro* 2016, 3(2):1–21

## O8 Emergence of disparity selective neurons through spike-based learning from naturalistic stereoscopic datasets

### Tushar Chauhan^1,2^, Timothée Masquelier^1,2^, Alexandre Montlibert^1,2^, Benoit R. Cottereau^1,2^

#### ^1^Université de Toulouse, Centre de Recherche Cerveau et Cognition, Toulouse, France; ^2^Centre National de la Recherche Scientifique, Toulouse Cedex, France

##### **Correspondence:** Tushar Chauhan (Tushar.Chauhan@cnrs.fr)

*BMC Neuroscience* 2017, **18(Suppl 1)**:O8

Models such as sparse coding [1] have shown that natural scene statistics can be used to predict basis units with Gabor-like receptive fields close to those observed in V1 simple-cells. Since the inputs to these models are natural images captured using a single camera, their outputs are monocular. Recently, attempts have also been made to exploit statistics of stereo-images of natural scenes [2]. The resulting bases units show binocular, Gabor-like receptive fields with population statistics close to those observed in V1. Although these models are able to replicate certain aspects of V1 binocular populations, they are either supervised, or mimic the result of learning from natural datasets, but not the process.

We propose a novel method of deriving monocular and binocular units through unsupervised learning from natural stereoscopic datasets using spike-timing-dependent-plasticity (STDP). Using the Hunter-Hibbard dataset [2], we first employed ON/OFF-center difference-of-Gaussian convolutions to mimic LGN responses (Fig. 1a). The responses were thresholded and converted to spike-latencies using a monotonically decreasing function. This ensured that the most activated units fired first, while the less active units fired late, or not at all. We then trained an STDP based neural network using 1 × 1 degree spatial pools from the aforementioned LGN layer. The network was composed of integrate-and-fire neurons and incorporated a lateral inhibition scheme. Finally, we characterized the receptive field in each eye by fitting Gabor functions.

Our results (Fig. 1b) showed that most units developed Gabor-like receptive fields similar to those observed in V1 simple cells, with a continuum of ocular dominance from pure monocularity to perfect binocularity. In line with single-unit recordings in primates, disparity selectivity was principally observed along the horizontal dimension, where it ranges between −0.5° and 0.5°. Neurons also showed selectivity to vertical disparity, although it was less pronounced. When tested with phase-shifted sine gratings, the units also showed disparity-tuning curves similar to those observed in the cat visual system.
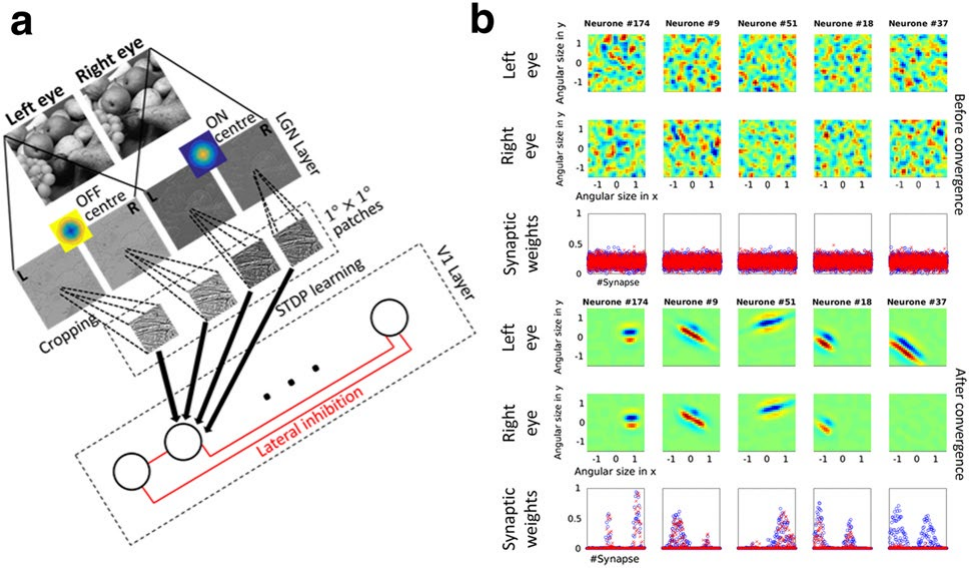


**Fig. 1 a** Schematic of the processing pipeline. **b** Five representative neurons (one per column) before and after convergence. Rows 1, 2: Left and right eye receptive fields before convergence; Rows 4, 5: The corresponding receptive fields after convergence; Rows 3, 6: Weights before and after convergence

**References**

1. Olshausen B, Field D. Emergence of simple-cell receptive field properties by learning a sparse code for natural images. *Nature* 1996, 381(6583):607–609.

2. Hunter D, Hibbard P: Distribution of independent components of binocular natural images. *J. Vis.* 2015, 15(13):6.

## O9 Cortical correlations support optimal sequence memory

### Moritz Helias^1,2^, Jannis Schuecker^1^, David Dahmen^1^, Sven Goedeke^1^

#### ^1^Institute of Neuroscience and Medicine (INM-6) & Institute for Advanced Simulation (IAS-6), Jülich Research Centre, Jülich, Germany; ^2^Department of Physics, Faculty 1, RWTH Aachen University, Aachen, Germany

##### **Correspondence:** Moritz Helias (m.helias@fz-juelich.de)

*BMC Neuroscience* 2017, **18(Suppl 1)**:O9

The brain processes time-varying input, but is it not known if its dynamical state is optimal for this task. Indeed, recurrent and randomly coupled networks of rate neurons display rich internal dynamics near the transition to chaos [1], which has been associated with optimal information processing capabilities [2, 3, 4]. In particular, the dynamics becomes arbitrarily slow at the onset of chaos similar to ‘critical slowing down’. The interplay between time-dependent input signals, network dynamics, and the resulting consequences for information processing are, however, yet poorly understood.

We here present a completely solvable model that allows us to investigate the effect of time-varying input on the transition to chaos. We analytically obtain the phase diagram spanned by the coupling strength and the input amplitude: External drive shifts the transition to chaos to significantly larger coupling strengths than predicted by linear stability analysis. The intermediate regime is absent in time-discrete networks [5] and only exists in their more realistic time-continuous counterparts. This novel dynamical regime combines locally expansive dynamics with asymptotic stability. We investigate sequential memory [5] and analytically show that memory capacity is optimal within the novel regime. Because it is unclear if cortex operates in such a computationally beneficial regime, we develop a finite-size mean-field theory which relates the statistics of measured covariances to the statistics of connections, in particular the spectral radius of the connectivity matrix. The theory shows that the large dispersion of spike count covariances across pairs of neurons, observed in massively parallel recordings, is an indicator that cortex indeed operates close to the breakdown of linear stability (see Fig. 1).
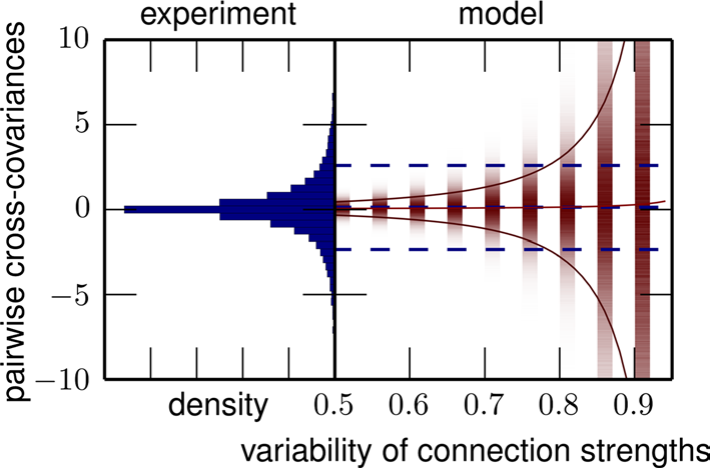


**Fig. 1** Distribution of spike count cross-covariances across neurons in macaque motor cortex. The low mean and large standard deviation (*blue dashed horizontal lines*) of experimentally observed cross-covariances between spike counts (*left*) are explained by a model network (*right*, shading indicates density of histogram) with a large spectral radius (R ~ 0.9) of the connectivity matrix.* Red curves*: analytical prediction for mean and ±1 standard deviation. Data from 155 neurons mostly located in layer 5 of macaque motor cortex (M1). Data courtesy of A. Riehle and T. Brochier

**Acknowledgements**

Helmholtz association: VH-NG-1028, Portfolio theme SMHB, and Jülich Aachen Research Alliance (JARA); European Union’s Horizon 2020 programme, grant agreement No. 720270. S.G., J.S., and D.D. contributed equally to this work.

**References**

1. Sompolinsky H, Crisanti A, Sommers HJ: Chaos in random neural networks. *Phys Rev Lett* 1988, 61: 259

2. Bertschinger N, Natschläger T: Real-time computation at the edge of chaos in recurrent neural networks. *Neural Comput* 2004, 16: 1413

3. Sussillo D and Abbott LF: Generating coherent patterns of activity from chaotic neural networks. *Neuron* 2009 63: 544

4. Toyoizumi T, Abbott LF: Beyond the edge of chaos: amplification and temporal integration by recurrent networks in the chaotic regime. *Phys Rev E* 2011, 84: 051908

5. Molgedey L, Schuchhardt J, Schuster H: Suppressing chaos in neural networks by noise. *Phys Rev Lett* 1992, 69: 3717

## O10 Rats decisions flexibly integrate sensory information and recent history of outcomes

### Alexandre Hyafil^1,2^, Ainhoa Hermoso-Mendizabal^1^, Pavel E. Rueda-Orozco^3^, Santiago Jaramillo^4^, David Robbe^5^, Jaime de la Rocha^1^

#### ^1^Idibaps, Barcelona, 08036, Spain; ^2^Center for Brain and Cognition, Barcelona, 08001, Spain; ^3^Universidad Nacional Autónoma de Mexico, Mexico City, Mexico; ^4^Institute of Neuroscience, University of Oregon, Eugene, Oregon, USA; ^5^Inmed, Marseille, 13000, France

##### **Correspondence:** Alexandre Hyafil (alexandre.hyafil@gmail.com)

*BMC Neuroscience* 2017, **18(Suppl 1)**:O10

Animal decisions not only reflect current sensory information but are also shaped by recent experience. There is however little understanding about the determinants of these history-dependent decision biases. We used rats in a novel two-alternative forced choice auditory discrimination task, in which the probability to repeat the previous stimulus category was varied in blocks of trials. Rats adapted to this environment by developing a strategy that capitalized on the serial correlations of the stimulus sequence: a bias towards repeating the same response built up after correct repetitions, and conversely an alternation bias developed after correct alternations. Strikingly, both repetition and alternation biases disappeared after an incorrect trial, irrespective of the number of previous correct trials performed previously.

A GLM analysis revealed that rat decisions in each trial relied on: (1) the current sensory stimulus; (2) a lateral bias towards (away from) the side of recently rewarded (unrewarded) responses on the last 5–10 trials, i.e. win-stay-lose-switch strategy; (3) a novel and strong transition bias that reinforced recent correct transitions (repetitions vs. alternations). Intriguingly the transition bias had no impact on choice after error trials. Subsequent analysis showed that the value of the bias was not reset but simply ignored after an error, and it was recovered after the first subsequent correct trial. Thus, the weight of the history-dependent transition bias could be flexibly and transiently put aside after error choices when possibly the reliability of the internal model was questioned. This nonlinear effect could not be captured by the GLM fitted to both correct and incorrect trials and was not present on the lateral bias, i.e. it was specific of the transition bias. We thus built a latent generative model of rat decisions, whereby lateral and transitions biases are updated at each trial, while the influence of the latter on current decisions is gated by a reward-dependent confidence signal. When fitted to the data, the model accounted quantitatively for all described behavioral effects: in particular, the absence of a transition bias after incorrect choices was due to a reset of the confidence signal. Because the value of the transition bias did not reset after errors but it kept the information about whether the animal would repeat or alternate, a single correct trial was sufficient to increase the confidence and recover the accumulated choice bias. Overall, we show that history-dependent biases in rodent perceptual choices reflect consistent strategic adaptations to behavioural outcomes.

## O11 Nicotinic modulation of hierarchal inhibitory circuit control over resting state ultra-slow fluctuations in the prefrontal cortex: modeling of genetic modification and schizophrenia-related pathology

### Marie Rooy^1^, Fani Koukouli^2,3^, David DiGregorio^3,4^, Uwe Maskos^2,3^, Boris Gutkin^1,5^

#### ^1^Group for Neural Theory, Laboratoire de Neurosciences Cognitives, INSERM Unité 969, Département d’Études Cognitive, École Normale Supérieure, Paris, France; ^2^Institut Pasteur, Neurobiologie intégrative des systèmes cholinergiques, Paris, France; ^3^CNRS UMR 3571, Paris, France; ^4^Institut Pasteur, Dynamic Neuronal Imaging, Paris, France; ^5^Centre for Cognition and Decision Making, National Research University Higher School of Economics, Moscow, Russia

##### **Correspondence:** Marie Rooy (rooy.marie@gmail.com)

*BMC Neuroscience* 2017, **18(Suppl 1)**:O11

The prefrontal cortex (PFC), key for higher order cognitive processes, exhibits spontaneous activity that is altered in schizophrenia [1]. Cortical acetylcholine (ACh) release modulates PFC activity via nicotinic acetylcholine receptors (nAChRs) [2] specifically expressed within a hierarchical circuit of inhibitory neurons within layer II/III [3]. Parvalbumin (PV) interneurons, expressing α7 nAChRs subunits [2], target pyramidal cells axosomaticaly, exerting divisive effects on their activity. Somatostatin (SOM) interneurons, expressing both α7 and β2 nAChRs subunits [2], target the dendrites of pyramidal cells, exerting substractive inhibition [4]. The α5 nAChRs subunits are expressed only by vasoactive intestinal polypeptide (VIP) interneurons, that preferentially inhibit the SOM cells. In vivo two-photon imaging showed that neural activity of PFC in mice is characterized by synchronous ultra-slow fluctuations, with alternating periods of high and low activity [5]. Genetic deletion of specific nAChRs subunits disrupted these ultra-slow fluctuations, leading to changes in synchrony and duration of activity states. Furthermore, mice expressing a human polymorphism in the α5 nAChRs subunits (α5SNP) associated with high risk for nicotine addiction and schizophrenia [6, 7], show reduced spontaneous activity in the PFC that is reversed by nicotine [3]. Using a circuit modeling approach, we studied the roles of distinct GABAergic interneurons in the generation of synchronous ultra-slow fluctuations. In order to study the effects of substractive vs. divisive inhibition on bistable dynamics in the pyramidal neuron, by the SOM and PV interneuron populations respectively, we used population firing rate modelling incorporating both mechanisms [8], and simulated the effects of nAChRs knock outs. With our model, we could fully account for the changes seen in resting state dynamics under the genetic modifications. We further predict that SOM interneurons play dominant role in the changes of activity-state structure seen in mutant mice, and in the restauration of activity to basal levels recorded in α5SNP mice under nicotine application.

**Acknowledgements**

This work was funded by RNF grant # 17-11-01273.

**References**

1. Barch, DM, Carter CS, Braver TS, Sabb FW, MacDonald A 3rd, Noll DC, Cohen JD: Selective deficits in prefrontal cortex function in medication-naive patients with schizophrenia. *Arch. Gen. Psychiatry* 2001, 58:280–288.

2. Bloem B, Poorthuis RB, Mansvelder HD: Cholinergic modulation of the medial prefrontal cortex: the role of nicotinic receptors in attention and regulation of neuronal activity. *Front. Neural Circuits* 2014, 8:17.

3. Koukouli F, Rooy M, Tziotis D, Sailor KA, O’Neill HC, Levenga J, Witte M, Nilges M, Changeux J-P, Hoeffer CA, Stitzel JA, Gutkin BS, DiGregorio DA, Maskos U: Nicotine reverses hypofrontality in animal models of addiction and schizophrenia. *Nature Medicine* 2017, 23:347–354.

4. Jadi M, Polsky A, Schiller J, Mel BW: Location-dependent effects of inhibition on local spiking in pyramidal neuron dendrites. *PLoS Comput Biol* 2012, 8(6):e1002550.

5. Koukouli F, Rooy M, Changeux J-P, Maskos U: Nicotinic receptors in mouse prefrontal cortex modulate ultraslow fluctuations related to conscious processing, *PNAS* 2016, 113(51):14823–14828.

6. Tobacco and genetics consortium: Genome-wide meta-analyses identify multiple loci associated with smoking behavior. *Nat. Genet* 2010, 42:441–447.

7. Schizophrenia working group of the psychiatric genomics consortium: Biological insights from 108 schizophrenia-associated genetic loci. *Nature* 2014, 511:421–427.

8. Chance FS, Abbott LF: Divisive inhibition in recurrent networks. *Network* 2000, 11(2):119–129.

## O12 The minimalistic mathematical model of the cerebral blood flow effects during cortical spreading depression

### Andrey Yu Verisokin^1^, Darya V Verveyko^1^, Dmitry E Postnov^2^

#### ^1^Department of Theoretical Physics, Kursk State University, Kursk, 305000, Russian Federation; ^2^Department of Physics, Saratov State National Research University, Saratov, 410012, Russian Federation

##### **Correspondence:** Andrey Yu Verisokin (ffalconn@mail.ru)

*BMC Neuroscience* 2017, **18(Suppl 1)**:O12

Cortical spreading depression (CSD) is one of the most common abnormalities in biophysical brain functioning. We have proposed a minimalistic model that reproduces the main dynamical features of cortical spreading depression dynamics and takes into account CSD and cerebral blood flow (CBF) coupling. Despite the fact that there are many mathematical models describing the CSD, most of them do not take into consideration the role of redistribution of CBF. In contrast to previous modelling attempt [1] which was chosen as the template, we focus on the role of CBF redistribution during the formation and propagation of wave front.

The flowchart of the developed model is shown in Fig. 1. The model includes six dynamical variables: activator v and inhibitor w, extracellular potassium z, blood vessel radius r and upstream blood pressure p, available neuron energy u (see Fig. 1).

The main model features:

1) we have modified and extended the components of basic model [1] that stand for the energy balance;

2) the proposed model counts the relation between the extracellular potassium concentration and the radius of the nearby located blood vessel: we take into account the effect of spatial coupling (functional hyperemia) by means of weighted summation of vasodilatory “driving force” over some distance from neuron;

3) we propose a lumped description for hemodynamic spatial coupling, being the direct result of blood flow redistribution between different areas fed from the single upstream arterial vessel.

Basing on the results of the numerical simulation we can conclude that the proposed model:

1) shows qualitatively reasonable results comparing with the experimental data: the uncorrelated noise-induced firing at rest; the persistent neuronal depolarization during the “active” phase of CSD; the depressed state afterwards, when model medium temporary losses the excitability and does not response on noisy stimuli;

2) reproduces main spatial patterns known for cortical spreading depression, migraine waves and spreading depolarization events observed in stroke and brain injuries;

3) predicts the formation of stationary dissipative Turing-like structures, formed due to the substantially different type of spatial relation – tissue perfusion. The role of perfusion in the formation of the structures was elucidated.
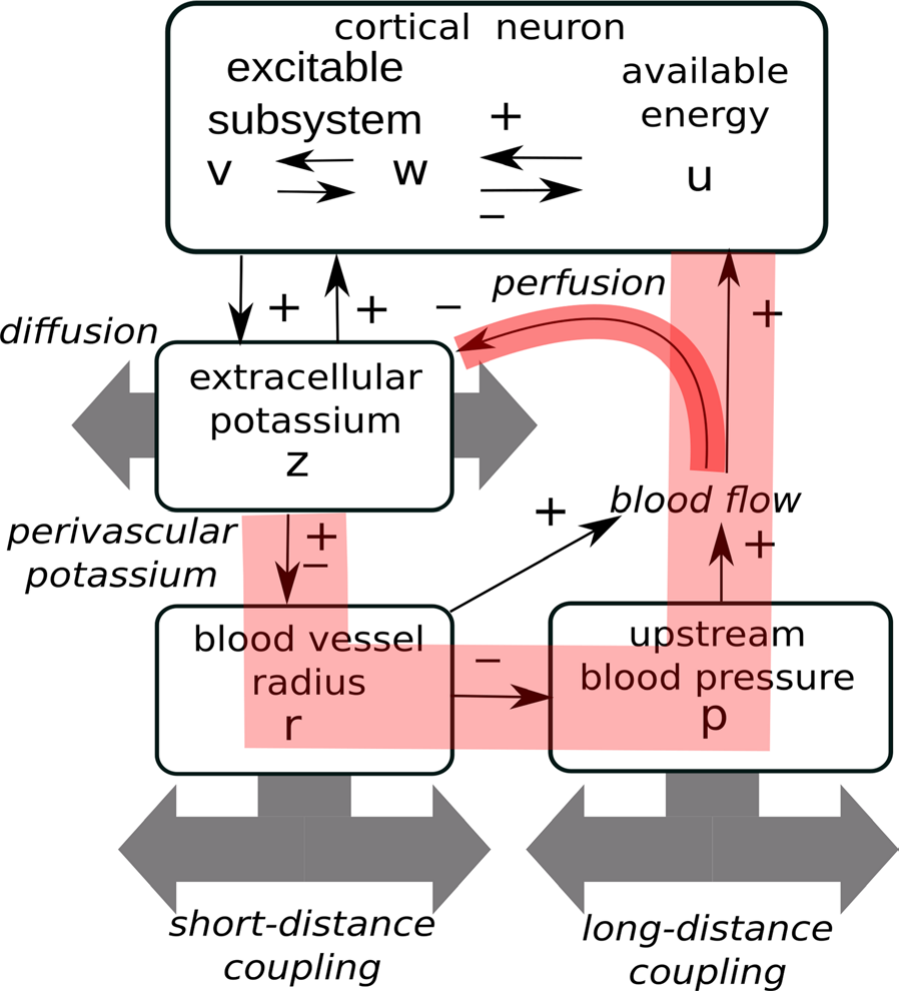


**Fig. 1** The schematic representation of flowchart of the developed model

**Acknowledgements**

This work is partially supported by RFBR grant 16-08-01203.

**Reference**

1. D E Postnov, D D Postnov, L Schimansky-Geier: Self-terminating wave patterns and self-organized pacemakers in a phenomenological model of spreading depression. *Brain Res.* 2012, 1434:200–211.

## O13 Necessity for coherence in motor control

### Willy Wong^1,2^, Omid Talakoub^3^, Robert Chen^4,5^, Milos Popovic^2,6^

#### ^1^Dept. of Electrical and Computer Engineering, University of Toronto, Toronto, ON, M5S3G4, Canada; ^2^Institute of Biomaterials and Biomedical Engineering, University of Toronto, Toronto, ON, M5S3G9, Canada; ^3^Dept. of Biology, York University, Toronto, ON, M3J1P3, Canada; ^4^Krembil Research Institute - University Health Network, Toronto, ON, M5T2S8, Canada; ^5^Division of Neurology, Faculty of Medicine, University of Toronto, Toronto, ON, M5S1A1, Canada; ^6^Toronto Rehabilitation Institute - University Health Network, Toronto, ON, M4G3V9, Canada

##### **Correspondence:** Willy Wong (willy.wong@utoronto.ca)

*BMC Neuroscience* 2017, **18(Suppl 1)**:O13

The basic premise of this study is that the coherence of neural activity is required for the coordination of motor control. Motor control involves a number of brain centres, most notably the cortex, the basal ganglia, thalamus and cerebellum. How messages are coordinated between the different centres during complex movements is an open question. Following [1], we examined the hypothesis that different neural sub-populations follow a “communication via coherence” hypothesis. That is, for two neural sub-populations to communicate their activity must be coherent and therefore exhibit some form of mathematical synchronization. As detailed in [2], we performed deep-brain measurements on patients undergoing treatment for Parkinson’s disease (n = 6) and dystonia (n = 7). During a brief period after implantation of the electrodes, we were able to record the activity from either the sub-thalamic nucleus (STN) or the globus pallidus interna (GPi). These recordings constitute local field potential (LFP) recordings. Patients were asked to perform one cycle of wrist movement lasting approximately one second in duration. The movements were executed either as externally cued or through self-initiation. Simultaneous to local field recordings measured at either the STN or GPi, electroencephalographic signals (EEG) were recorded over the motor cortex. For LFP, recordings were processed by subtracting the activity from adjacent electrodes. EEG was recorded in a bipolar montage (either C3-Cz or C4-Cz). We believe that the activity we record is local in origin and not due to volume conduction, or due to the use of a common reference.

Our results show that during movement, and only during movement, is there significant coupling between changes in the power of the activity with changes in coherence between the basal ganglia and the motor cortex. The changes can happen such that for beta band activity (20–30 Hz) both power/coherence is high pre and post movement, but low during course of movement. For gamma activity (30+ Hz), we observe the opposite: only during movement do we observe a coupling of increased power with increased levels of coherence either between GPi-cortex or STN-cortex. The coupling of power with coherence is not artifactual.

To better understanding the origins of these findings, we need to develop suitable mathematical models of coupled neural ensembles. We have been extending the Kuramoto model of coupled oscillators for application to this problem. Two distinct neural ensembles (in the basal ganglia and in the cortex) have neurons that are each interconnected. Moreover, the two ensembles are further connected to each other through additional links. What we can show is that an increase in power in either ensembles will lead to increased amplitude/phase coherence between the two ensembles just as found experimentally. This thus provides a first model of motor coordination between cortex and basal ganglia. Establishing the necessity for coherence in motor coordination suggests new strategies for neuromodulation similar to how functional electrical stimulation works to restore peripheral motor function.

**Acknowledgements**

This work was supported by a Canadian Institutes of Health Research grant to RC, Dean Connor and Maris Uffelmann Donation to MP, Toronto Rehab Foundation to MP, and grants from the Natural Science and Engineering Research Council of Canada to MP and WW.

**References**

1. Fries P. A mechanism for cognitive dynamics: neuronal communication through neuronal coherence. *Trends Cogn Sci* 2005;9(10):474–480.

2. Talakoub O, Neagu B, Udupa K, et al. Time-course of coherence in the human basal ganglia during voluntary movements. *Sci Rep* 2016;6:34930.

3. Kuramoto Y. Self-entrainment of a population of coupled non-linear oscillators In: Araki, H, editor. *International Symposium on Mathematical Problems in Theoretical Physics.* Springer 1975. p. 420–422

## O14 Dissecting gamma phase and amplitude-specific information routing in V4 of macaque during selective attention

### Dmitriy Lisitsyn^1^, Eric Drebitz^2^, Iris Grothe^3^, Sunita Mandon^2^, Andreas Kreiter^2^, Udo Ernst^1^

#### ^1^Computational Neuroscience Lab, Institute for Theoretical Physics, Bremen University, Bremen, Germany; ^2^Institute for Theoretical Neurobiology, Brain Research Institute, Bremen University, Bremen, Germany; ^3^Ernst Strüngmann Institute (ESI) for Neuroscience, Frankfurt, Germany

##### **Correspondence:** Dmitriy Lisitsyn (dmitriy@neuro.uni-bremen.de)

*BMC Neuroscience* 2017, **18(Suppl 1)**:O14

Communication through coherence (CTC) postulates that stimulus information transmission is enhanced between oscillating neural populations in a favorable phase relationship, and suppressed otherwise. For example, in the case of visual cortical gamma-band synchronization during selective attention, V1 spikes arriving to V4 during its excitability peaks should be much more likely to elicit further spikes, resulting in effective signal gating; V1 spikes arriving during excitability troughs should fail or at least be less effective in evoking further activity. Further, it has been observed that average gamma power increases with attention, however, this increase appears to occur in bursts, rather than a constant oscillation. If the CTC hypothesis holds, one should expect descriptive gamma phase and amplitude dependent modulations in stimulus information routing in V4.

To explore this idea, we analyzed neural data from a previous study [1], recorded from V4 superficial layers in macaques performing a visual spatial attention task. The task required the animals to attend one of two dynamic stimuli over an extended time period. Crucially, each stimulus was superimposed with its own fluctuating luminance signature, irrelevant to the behavioral task. This allowed us to quantify the information content *I* of each stimuli conveyed by the physiological signal, by computing spectral coherence between each stimuli’s luminance signal and V4 activity. To assess modulation effects at multiple population scales, we analyzed both LFP and spiking activity. Using gamma-band activity extracted from LFP, we dissected both LFP and spiking neural activity into phase/amplitude-specific components. We then computed the information contribution of each stimulus to these components, giving us the opportunity to assess phase/amplitude signal gating effects.

The results show that information routing is modulated by the gamma phase for both LFP and spiking activity. In LFP, we found the information routing at excitability peaks *I*_peak_ is significantly higher than at excitability troughs *I*_trough_ for both attended and non-attended stimuli (Fig. 1a). We did not see this effect for spikes, which still show significant gamma phase dependence but without a preference for a specific phase across recording sessions. Comparing the stimuli content during high gamma activity *I*_high_γ against low gamma activity *I*_low_γ, we found that the spiking activity exhibits significant gating increase for the attended stimulus and decrease for the non-attended stimulus (Fig. 1b), however, we do not find this effect in the LFP.

In summary, our study confirms basic predictions on the nature of selective information processing, namely its modulation in dependence on phase and amplitude of LFP gamma activity. Surprisingly, consistent phase modulation was only found in LFPs, while consistent amplitude modulation was only seen in spiking activity, indicating that the mechanisms implementing CTC are not yet fully understood. In particular, our results strongly motivate a refinement of current CTC models, requiring an approach encompassing different levels of complexity capable of reproducing local spiking and global population activity from different laminar sources.
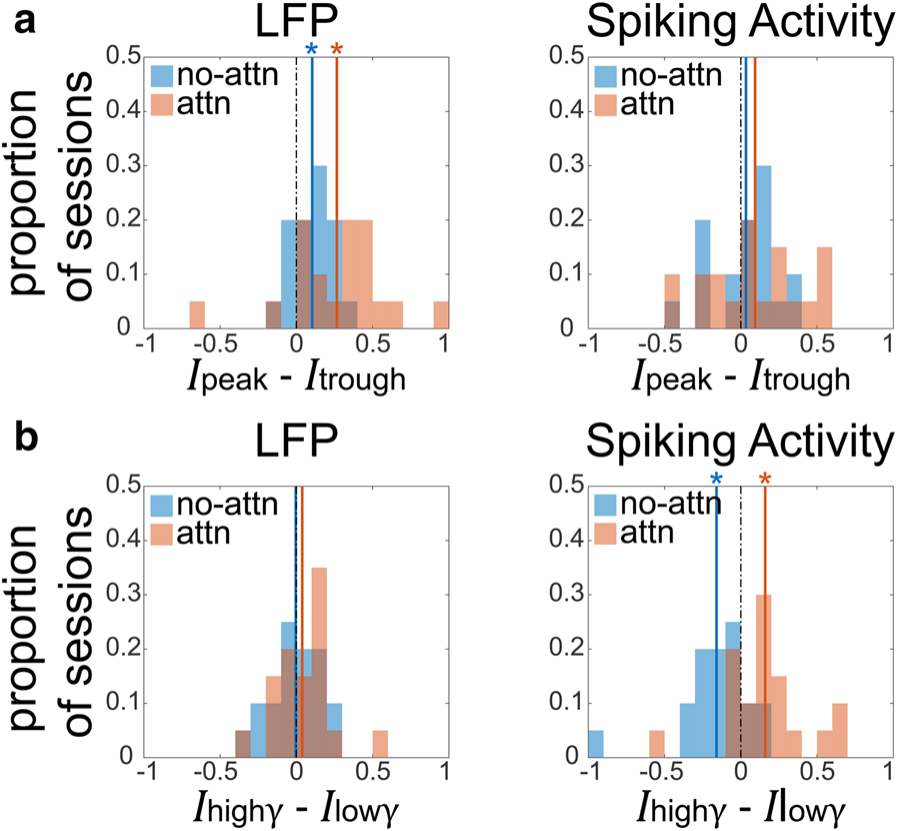


**Fig. 1 a** Peak vs trough γ phase info routing **b** high vs low γ amplitude info routing

**Acknowledgements**

This work was supported by the DFG priority program SPP 1665 (ER 324/3-1).

**Reference**

1. Grothe I, Rotermund D, Neitzel SD, Mandon S, Ernst UA, Kreiter AK, Pawelzik KR. Attention selectively gates afferent signal transmission to area V4. *bioRxiv*. 2015, 019547.

## O15 Structure-Function Relationships via Neural Field Theory

### Peter A. Robinson^1,2^, Xuelong Zhao^1,2^, Kevin M. Aquino^1,2,3^, John D. Griffiths^1,2,4^, Grishma Mehta-Pandejee^1,2^, Natasha Gabay^1,2^, James MacLaurin^1,2^, and Somwrita Sarkar^1,2,5^

#### ^1^School of Physics, University of Sydney, Sydney, NSW 2006 Australia; ^2^Center for Integrative Brain Function, University of Sydney, Sydney, NSW 2006, Australia; ^3^Sir Peter Mansfield Imaging Center, University of Nottingham, Nottingham, UK; ^4^Rotman Research Institute at Baycrest, Toronto, Ontario, Canada; ^5^Design Lab, School of Architecture, University of Sydney, Sydney, NSW 2006, Australia

##### **Correspondence:** Peter A. Robinson (peter.robinson@sydney.edu.au)

*BMC Neuroscience* 2017, **18(Suppl 1)**:O15

Patterns of brain activity are observed to be highly conserved across states of arousal, and between task and non-task conditions. This strongly suggests that these are natural modes (eigenmodes) of the brain, which are excited in different ways under different circumstances. Neural field theory (NFT), which averages over brain microstructure, is ideally suited to deriving brain eigenmodes and interpreting them in terms of underlying physiology. It also provides means of systematically interrelating structure and function via these eigenmodes.

Here, NFT is used to predict the eigenmodes of the continuous cortical surface, including interhemispheric connections. For comparison, eigenmodes of a discrete cortical connection matrix are calculated by standard matrix procedures. Mode energies and symmetry properties are used to constrain interhemispheric conductivities and physiological properties of the cortex. Eigenmodes are then used to derive underlying effective and functional connectivities from system transfer functions and two-point correlations of background activity, respectively.

Neural field eigenmodes are shown to occur in a hierarchy closely related to that of the eigenmodes of a sphere, with added symmetries induced by bihemispheric structure. A close correspondence is also found with the eigenmodes of an anatomical connection matrix, confirming the validity of the neural field approach. The results demonstrate that the brain is in a near-critical state, consistent with estimates from electroencephalographic spectra. It is found that each hemisphere receives near-balanced inputs, with approximately 15% of net inputs coming from the contralateral hemisphere, 73% from the ipsilateral one, and 12% from the environment, meaning that it is in a highly introspective state. Most activity is predicted to be in symmetric modes, in accord with experiment.

NFT allows structure and activity to be unequivocally interrelated, including the correlations used to define functional connectivity matrices. Eigenmode decomposition of these matrices enables underlying effective connectivities to be systematically derived from functional connectivities, and vice versa, and related to resulting activity patterns. This means that relatively easily observed correlations can be used to infer both average structure and the strengths of effective connectivities that it supports in a noninvasive manner.

In summary, physiologically-based NFT thus explains and unifies multiple phenomena relating to structure, function, and activity via eigenmodes. This allows analysis of activity and structure in terms of the natural dynamic modes of the system, rather than ones that are defined via statistical signal analyses that do not incorporate physiology.

**Acknowledgements**

This work was supported by the Australian Research Council under Grants CE140100025 and LF140100007.

## O16 Dynamic Operations of Hierarchically Interacting Canonical Microcircuits

### Tim Kunze^1,2^, Jens Haueisen^2^, and Thomas R. Knösche^1^

#### ^1^Max Planck Institute for Human Cognitive and Brain Sciences, Leipzig, Germany; ^2^Institute of Biomedical Engineering and Informatics, Ilmenau University of Technology, Ilmenau, Germany

##### **Correspondence:** Tim Kunze (tkunze@cbs.mpg.de)

*BMC Neuroscience* 2017, **18(Suppl 1)**:O16

Research on canonical microcircuits conceptualizes findings of the recursive occurrence of neural populations and coupling patterns in vertically and horizontally structured divisions (i.e. cortical columns) of the cerebral cortex [1]. The profound description and examination of the link between canonical architectures and the associated functionality promises a better understanding of higher level functions which emerge from the interaction of canonical microcircuits. Fundamental for this interaction is the embedding canonical microcircuits in hierarchical networks [2], mediating both bottom-up and top-down signals to specific neuronal populations. Here, computational studies can help to formulate hypotheses about constitutive mechanisms, which are experimentally identifiable in the neural substrate.

We use a neural mass model [3], where a pyramidal cell population (Py) receives negative feedback from an inhibitory interneuron population (IIN) and positive feedback via a secondary excitatory population of interneurons (EIN), representing neurons in layer IV. We systematically apply transient afferent inputs, modeled by pulses of various magnitude and duration, as bottom-up signals to the EIN or as top-down signals to the Py [2] and monitor the behavior of the Py. These response behaviors are classified as: a) *nonresponsive* for sub-threshold transient deflections, b) *transfer* for supra-threshold transient deflections, and c) *memory* for sustained supra-threshold deflections and are mapped to the stimulation parameter range.

Single-channel stimulations, either bottom-up (to EIN) or top-down (to Py), lead to differential response behaviors, where strong and long bottom-up stimulations are preferably stored (memory behavior), in contrast to top-down signals, which predominantly show transient deflections. In a concomitant stimulation, constant top-down input modulates the model’s sensitivity to pulsed bottom-up stimulation in favor of the memory response behavior. We employ this modulatory influence in a hierarchical network (Fig. 1a) comprising two canonical microcircuits to show a conceivable neural mechanism for the dynamic adaptation of a perceptual threshold. In this configuration, a target stimulus is not able to excite a perceptual area, unless a priming stimulus tunes the network’s sensitivity.

The differential response behaviors to top-down and bottom-up stimuli indicate the functional role of separate input channels in canonical microcircuits. Exemplarily, we show one constitutive operation emerging from interacting microcircuits, but expect many more mechanisms relevant in cognitive disciplines like language or memory, such as stimulus selection or structure building computations. Further, the present results in the hierarchical setup demand a further evaluation in light of predictive coding where important findings of neural communication have been put forward.
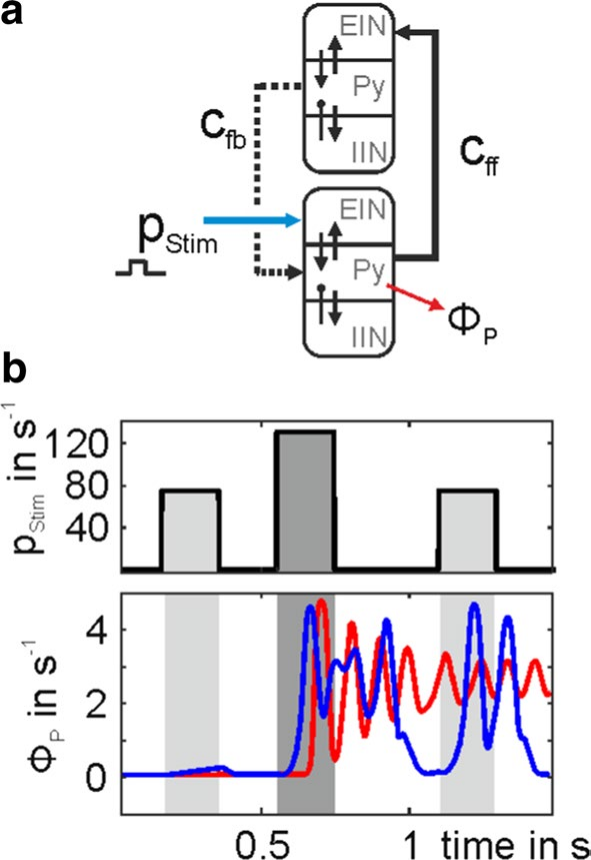


**Fig. 1 a** Adaptive dynamical shift of a perceptual threshold in a hierarchical configuration of two interacting neural mass models, mimicking canonical microcircuits. **b** A bottom-up target input (*light grey*) excites the lower area only after previous application of a priming stimulus (*dark grey*)

**References**

1. Douglas JD, Martin KA: Mapping the Matrix: The Ways of Neocortex. Neuron 2007, 56:226–238.

2. Felleman DJ, Van Essen DC: Distributed hierarchical processing in the primate cerebral cortex. Cerebral Cortex 1991, 1:1–47.

3. Spiegler A, Kiebel SJ, Atay FM, Knösche TR: Bifurcation analysis of neural mass models: Impact of extrinsic inputs and dendritic time constants. *NeuroImage* 2010, 52:1041–1058

## O17 Learning structure of 3D objects with cortical columns

### Subutai Ahmad^1^, Yuwei Cui^1^, Marcus Lewis^1^ and Jeff Hawkins^1^

#### ^1^Numenta, Redwood City, CA 94063, USA

##### **Correspondence:** Subutai Ahmad (sahmad@numenta.com)

*BMC Neuroscience* 2017, **18(Suppl 1)**:O17

The neocortex is organized in cellular layers. Connections between layers run mostly perpendicular to the surface of the neocortex, which suggests a “columnar” pattern of activation across layers. The cells in some layers also send their axons across long distances parallel to the surface of the neocortex, which suggests a “laminar” pattern of activation across multiple columns. The vertical and horizontal spread of axons is a ubiquitous feature of all neocortical regions.

In this study, we propose a network model that utilizes both intra-column and cross-column connections for robust object learning and recognition (Fig. 1). The model consists of a set of cortical columns, where each cortical column processes a different subset of the sensory input space. An object consists of a set of component features at particular locations on the object. Each cortical column learns an object by forming feedforward connections from its component features to a set of active neurons in a different cellular layer. After learning, sensation of a sequence of object features leads to activations of the corresponding neural population representing the object.

Since features can be shared among multiple objects, information received by a single cortical column is often ambiguous. The model uses auto-associative connections to integrate many sensations over time and can converge onto unique object representations once sufficient feature are sampled. The recognition speed and accuracy can be improved by simultaneously considering multiple cortical columns with lateral connections, where each column learns feedforward connections independently and learns cross-column lateral connections according to Hebbian rules. The lateral inputs target distal dendritic segments. Although they are not strong enough to directly activate a neuron, neurons with both lateral input and feedforward input will fire earlier and prevent other neurons from responding [1]. The cross-columnar connections bias each column to form a representation that is consistent with the partial knowledge of all the interconnected columns. We show that objects can be recognized faster and that each cortical column can store more objects by using cross-column connections.

The model is consistent with a large body of anatomical and physiological evidence and provides a number of predictions that can be tested in future experiments.
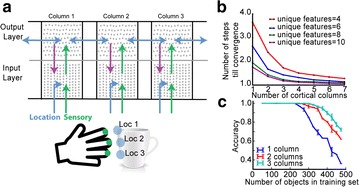


**Fig. 1 a** We consider the problem of object recognition with a set of cortical columns. Each column receives sensory input from a different sensor (e.g., different finger). A first layer of the network transforms the raw sensory input into sparse distributed representations that corresponds to object features. The second layer receives feedforward inputs from the first layer. It recognizes an object by converging onto a stable activation pattern through lateral connections. **b** The recognition speed increases as a function of column number. **c** Retrieval accuracy of object during testing vs. the number of learned objects. More objects can be learned with networks with more cortical columns

**Reference**

1. Hawkins J, Ahmad S: Why neurons have thousands of synapses, a theory of sequence memory in neocortex. *Front Neural Circuits* 2016, 10:1–13.

## O18 Influence of network topology on spreading of epileptic seizure

### Simona Olmi^1^, Spase Petkoski^2^, Fabrice Bartolomei^3,^ Maxime Guye^4^, Viktor Jirsa^2^

#### ^1^Weierstrass Institute, Mohrenstr. 39, 10117 Berlin, Germany; ^2^Aix Marseille Université, Inserm, Institut de Neurosciences des Systèmes, UMR S 1106, 13005, Marseille, France; ^3^Assistance Publique Hôpitaux de Marseille, Hôpital de la Timone, Service de Neurophysiologie Clinique, CHU, 13005, Marseille, France; ^4^Faculté de Médecine de la Timone, centre de Résonance Magnétique et Biologique et Médicale (CRMBM, UMR CNRS-AMU 7339), Medical School of Marseille, Aix-Marseille Université, 13005, Marseille, France

##### **Correspondence:** Simona Olmi (simona.olmi@gmail.com)

*BMC Neuroscience* 2017, **18(Suppl 1)**:O18

In partial epilepsy, seizures originate in a local network, the so-called epileptogenic zone, before recruiting other close or distant brain regions. Correctly delineating the epileptogenic and the propagation zone is essential for successful resective surgery. In particular, the stereotaxic EEG (SEEG) is used to edge the resection zone. Nevertheless, the propagation pathways of epileptic seizures are still largely unknown. We utilize a specific dynamical model for epilepsy, the Epileptor model [1], to predict the recruitment network given the seizure origins and the structural brain connectivity. Thus, we try to understand the role played by the topology in constraining the recruitment process and we suggest a paradigm for epileptic surgery that relies on minimal invasiveness and maximum effectiveness. In particular, we schematize the brain network dynamics in terms of neural mass models able to captures the details of the autonomous slow evolution of interictal and ictal phases; these mass models are coupled among them and the coupling terms model the effective presence of nerve pathways and fibers among different brain regions [2]. In this framework, it is possible to identify the minimal number of local disconnections of the epileptogenic zone that are necessary to stop seizure propagation via the application of linear stability analysis and, therefore, to define the optimal set of links to be cut in order to stop seizure propagation (see Fig. 1). In order to demonstrate the potential use of this framework in practice, we apply our methods to structural connectivity matrices derived from patients affected by partial epilepsy. In all cases a partial disconnection, that counts for the resection of few pathways, is sufficient to stop seizure activity in the brain. Therefore, we demonstrate that seizure spreading is thus supported and enhanced by the underlying topology and that a disconnection procedure, if well addressed, can become a fruitful procedure to improve the success rate of epilepsy surgery.
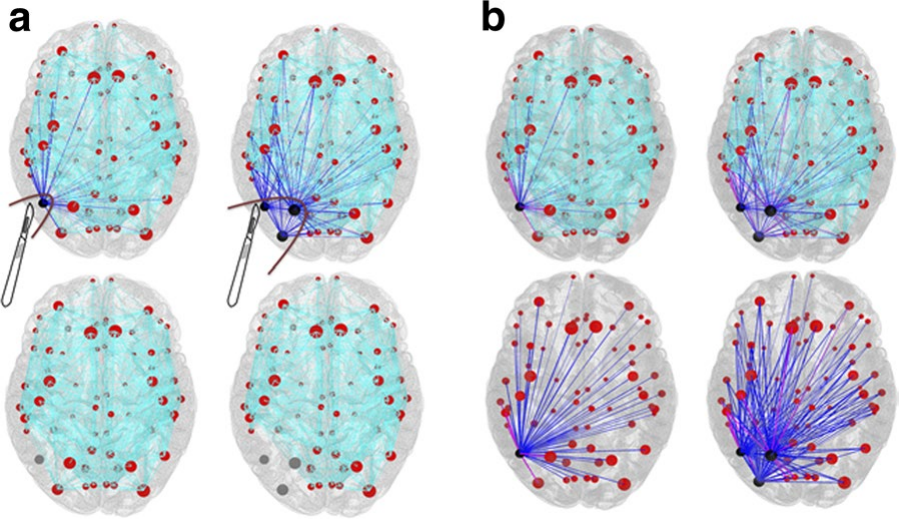


**Fig. 1 a** Standard resection technique, where the entire epileptogenic zone (EZ) is removed during surgical operation. Blue links represent the outgoing connections of the EZ and are completely removed during the current surgical procedures. **b** Lesioning depicts the minimal number of links that are sufficient to be removed (magenta) in order to stop the seizure, versus the total number of outgoing links from the EZs (blue) that are removed during the resection of an entire EZ. Cyan links represent in both panels the full connectivity of the network

**References**

1. Jirsa VK *et al.* : On the nature of seizure dynamics. *Brain* 2014, 137:2210–2230.

2. Proix T *et al.* : Permittivity Coupling across Brain Regions Determines Seizure Recruitment in Partial Epilepsy, *J. Neurosci.* 2014, 34: 15009–15021.

## O19 A model-based approach for detecting multiple change points in multivariate spike count data

### Hazem Toutounji, Daniel Durstewitz

#### Department of Theoretical Neuroscience, Bernstein Center for Computational Neuroscience, Central Institute of Mental Health, Medical Faculty Mannheim, Heidelberg University, Heidelberg, Germany

##### **Correspondence:** Hazem Toutounji (hazem.toutounji@zi-mannheim.de)

*BMC Neuroscience* 2017, **18(Suppl 1)**:O19

Neural data often consist of multiple single unit recordings in the form of spike count time series. These time series are often highly nonstationary, where statistical moments, such as firing rates, vary to potentially encode features of the experimental paradigm, like changes in external input or different task phases. Changes in the firing rates may be sudden or gradual, and their time scale and onset may reflect information regarding neural computations, such as learning [1] or the accumulation of sensory evidence [2].

Here we develop an approach for detecting and parametrising multiple changes in multivariate spike count data within the statistical framework of State Space Models (SSM) [3]. The model assumes a nonlinear, nonstationary, autoregressive Gaussian process that captures the underlying latent neural dynamics. However, given their discrete, nonnegative nature, assumptions of normality are not guaranteed to produce consistent estimates of spike count statistical moments. Instead, the Gaussian process generates spike counts by a Poisson observation function. Both latent trajectories in phase space and latent model parameters, in addition to observation model parameters, are estimated by a 3-stage Expectation-Maximisation (EM) procedure [4]. The latter relies on Newton’s method [5] to maximise, under constraints, a global Laplace approximation [6] of spike-count data’s log-likelihood, given the SSM and its parameters. The dimensionality of the latent model equals the number of unknown nonstationary events, termed change points, and is selected by a cross-validation procedure. Observations, on the other hand, are generally of a much higher dimension than the latent dynamics. Due to this substantial dimensionality reduction [7], latent trajectories, thus, offer a parsimonious representation of the most relevant features in neural dynamics.

The estimation procedure is first tested on simulated data, to assure that the latent states and model parameters are correctly identified in comparison to the ground truth. As a real data example, the model is fitted to multiple single unit recordings from rat medial prefrontal cortex neurons during an operant rule switching task. The resulting reconstruction of the underlying dynamics will allow matching the neural correlates of learning to their behavioral counterpart, by relating behavioral changes to population-wide change points, as estimated by the model.

**Acknowledgements**

The work was funded by the German Research Foundation (DFG) (SPP1665 / DU 354/8-1) and through the German Ministry for Education and Research (BMBF) via the e:Med framework (01ZX1314E). The authors thank Dr. Florian Bähner for providing the prefrontal cortex data.

**References**

1. Durstewitz D, Vittoz NM, Floresco SB, Seamans JK: Abrupt transitions between prefrontal neural ensemble states accompany behavioral transitions during rule learning. *Neuron* 2010, 48:438–448.

2. Latimer KW, Yates JL, Meister MLR, Huk AC, Pillow JW: Single-trial spike trains in parietal cortex reveal discrete steps during decision-making. *Science* 2015, 349(6244):184–187.

3. Durstewitz D, Koppe G, Toutounji H: Computational models as statistical tools. *Curr Opin Behav Sci* 2016, 11:93–99.

4. Dempster AP, Laird NM, Rubin DB: Maximum likelihood from incomplete data via the EM algorithm. *J R Stat Soc Ser B* 1977, 39:1–38.

5. Nocedal J, Wright SJ: *Numerical Optimization, 2nd Edition*. New York: Springer-Verlag; 2006.

6. Paninski L, Ahmadian Y, Ferreira DG, Koyama S, Rahnama RK, Vidne M, Vogelstein J, Wu W: A new look at state-space models for neural data. *J Comput Neurosci* 2010, 29(1–2):107–126.

7. Cunningham JP, Yu BM: Dimensionality reduction for large-scale neural recordings. *Nat Neurosci* 2014, *17*(11): 1500–1509.

## O20 Geppetto: an open source visualisation and simulation platform for neuroscience

### Matteo Cantarelli^1^, Adrian Quintana^2,4^, Boris Marin^4^, Matt Earnshaw^4^, Padraig Gleeson^4^, Robert Court^3^, Robert McDougal^6^, R. Angus Silver^4^, Salvador Dura-Bernal^5^, Stephen Larson^1^, William W. Lytton^5^, Giovanni Idili^1^

#### ^1^OpenWorm Foundation, Delaware, USA; ^2^EyeSeeTea Ltd., London, UK; ^3^Edinburgh University, Edinburgh, UK; ^4^Department of Neuroscience, Physiology and Pharmacology, University College London, London, UK; ^5^State University of New York Downstate Medical Center, Brooklyn, NY, USA; ^6^Yale University, New Haven, CT, USA

##### **Correspondence:** Matteo Cantarelli (matteo@openworm.org)

*BMC Neuroscience* 2017, **18(Suppl 1)**:O20

Geppetto (geppetto.org) is an open-source web-based platform to explore and simulate neuroscience data and models. The platform, originally designed to support the simulation of a cell-level model of *C. elegans* as part of the OpenWorm project [1], has grown into a generic framework suitable for various neuroscience applications, offering out of the box solutions for data visualisation, integration and simulation. Geppetto is today used by Open Source Brain (opensourcebrain.org) (Fig. 1a), to explore and simulate computational neuroscience models described in NeuroML version 2 with a variety of simulators and by the Virtual Fly Brain (virtualflybrain.org) (Fig. 1b) to explore and visualise anatomy (including neuropil, segmented neurons and gene expression pattern data) and ontology knowledge base of *Drosophila melanogaster*. Geppetto is also being used to build a new experimental UI for the NEURON simulation environment [2, 3] (Fig. 1c) based on *Python* and *Jupyter*. WormSim (wormsim.org) (Fig. 1d) embeds Geppetto to let users explore dynamic mechanical and electrophysiological models of *C. elegans* produced by the OpenWorm project. Geppetto is capable of reading and visualising experimental data in the *NWB* format (nwb.org) to allow experimental and computational neuroscientists to share and compare data and models using a common platform. Geppetto is freely available, well documented and has an active user community. Interested potential users can try out the latest version of the platform at live.geppetto.org.
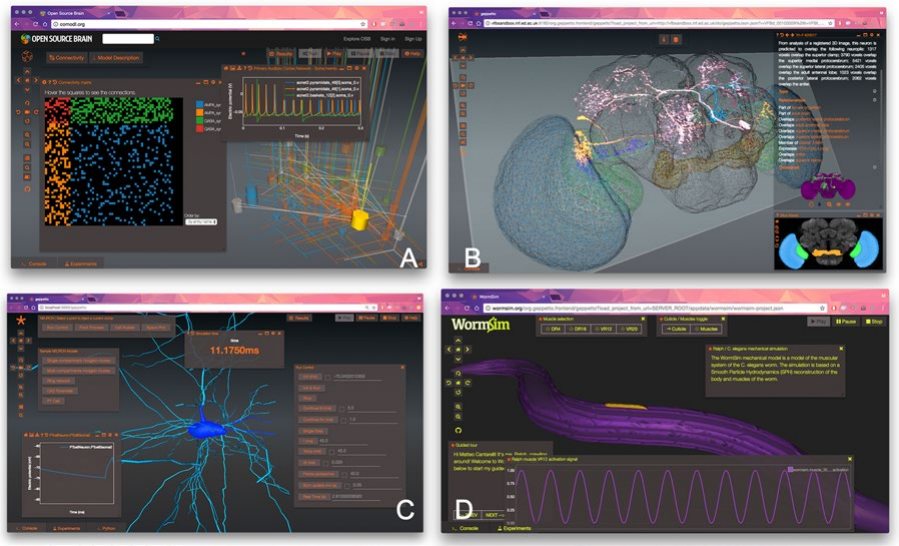


**Fig. 1** Geppetto in different deployments

**References**

1. Szigeti B, Gleeson P, Vella M, Khayrulin S, Palyanov A, Hokanson J, Currie M, Cantarelli M, Idili G, Larson S. OpenWorm: an open-science approach to modelling Caenorhabditis elegans. *Front. Comput. Neurosci.* 2014.

2. Carnevale, N.T. and Hines, M.L. The NEURON Book. Cambridge, UK: Cambridge University Press, 2006.

3. NEURON Experimental UI [https://github.com/MetaCell/NEURON-UI]

## O21 Position is coherently represented during flickering instabilities of place-cell cognitive maps in the hippocampus

### Lorenzo Posani^1^, Simona Cocco^1^, Karel Ježek^2^, Rémi Monasson^1^

#### ^1^Laboratories of Statistical & Theoretical Physics, Ecole Normale Supérieure, Paris, France; ^2^Laboratory of Experimental Neurophysiology, Biomedical Center, Charles University, Prague, Czech Republic

##### **Correspondence:** Lorenzo Posani (lorenzo.posani@ens.fr)

*BMC Neuroscience* 2017, **18(Suppl 1)**:O21

Place cells in hippocampus exhibit sharp spatially-related firing fields, which are formed when the animal explores new environments and are retrieved, as memories, each time the rat is placed back in those specific settings. Knowledge of the environment-specific set of place fields (map) allows for the application of Bayesian statistics to infer the position of the rodent from neuronal activity (Fig. 1b, c). Likewise, functional-connectivity models, based only on neural correlations, i.e. with no knowledge of place fields or position, can identify the expressed map as a function of time (Fig. 1a) [1]. We apply both these inference procedures to CA3 recordings from a recent “teleportation” experiment [2], in which instantaneous switches between the identity of two familiar environments trigger the instability of the recalled memory state, which flickers back and forth between the two corresponding maps (Fig. 1a, e). Our analysis shows that the rat position is not accurately inferred during the unstable periods, under conventional approach relying on brain processing only the external input information (i.e. environment cues, Fig. 1b, d: red curve). However, if the position is inferred using the template reflecting the decoded inner state of the network, the position error is significantly reduced, reaching values comparable to the stable conditions (Fig. 1c, d: blue curve). Results suggest that position is robustly encoded in CA3, even during periods of conflict or ambiguity in the input information resulting in global map changes on fast dynamical time scales.
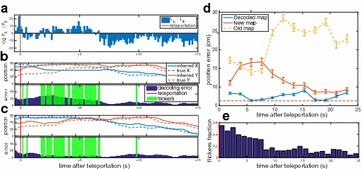


**Fig. 1**. Position inference during flickering instabilities. **a** Decoded environment (log-likelihood difference) as a function of time after a teleportation in CA3. Note the flickering dynamics in the 0–5 s interval. **b** Inferred vs. real positions of the animal; place fields corresponding to light conditions were used for the inference. Freely-moving rat in a 60 × 60 cm box. **c** Same as B with position inferred using the place fields associated to the *decoded map* (sign of ∆L in panel A). **d** Positional errors averaged over 15 teleportation events; dashed line indicates the level of error for stable conditions (no light switches). **e** Fraction of flickering time bins (∆L-decoded map differs from light conditions) as a function of time after the light switch

**Acknowledgements**

This work was partially supported by the HFSP RGP0057/2016 project.

**References**

1. Posani L, Cocco S, Jezek K, Monasson R: Functional connectivity models for decoding of spatial representations from hippocampal CA1 recordings. *Journal of Computational Neuroscience* 2017, 1–17

2. Jezek K, Henriksen EJ, Treves A, Moser EI, Moser MB: Theta-paced flickering between place-cell maps in the hippocampus. *Nature* 2011, 478.7368: 246–249.

###### Publisher’s Note

Springer Nature remains neutral with regard to jurisdictional claims in published maps and institutional affiliations.

